# Supplementary material for: Photon-phonon collaboratively pumped laser
Source: Nat Commun. 2023 Dec 7;14:8110. doi: 10.1038/s41467-023-43959-9 (PMC10703827; doi:10.1038/s41467-023-43959-9)
Supplement: Supplementary file 1 — Supplementary information [file 41467_2023_43959_MOESM1_ESM.pdf]

Supplementary Materials for

Photon-phonon collaboratively pumped laser

Yu Fu[1,#], Fei Liang[1,#], Cheng He[2], Haohai Yu[1,*], Huaijin Zhang[1, §], Yan-Feng Chen[2, †]

[1] State Key Laboratory of Crystal Materials and Institute of Crystal Materials, Shandong University, Jinan 250100, China

[2] National Laboratory of Solid State Microstructures & Department of Materials Science and Engineering, Nanjing University, Nanjing 210093, China

[#]These authors contributed equally: Yu Fu, and Fei Liang

[*]Corresponding author

haohaiyu@sdu.edu.cn

[§]Corresponding author

huaijinzhang@sdu.edu.cn

[†]Corresponding author

yfchen@nju.edu.cn

Outline

Section-I: Additional Figures and Tables

Section-II: Alternative explanations for Nd:YVO_4 laser

Section-III: Thermal effect in a four-energy level laser system

Section-IV: The difference between alexandrite laser and Nd:YVO_4 laser

Section-V: Selection rules of phonons in electron-phonon coupling process

Section-VI: Theory for general threshold equation $P_{th} = C/T_{th}$

Section-VII: Steady-state rate equation for PPCP laser

Section-VIII: A comparison between solid-state lasers, semiconductor lasers and PPCP laser.

Supplementary References

Section-I: Additional Figures and Tables

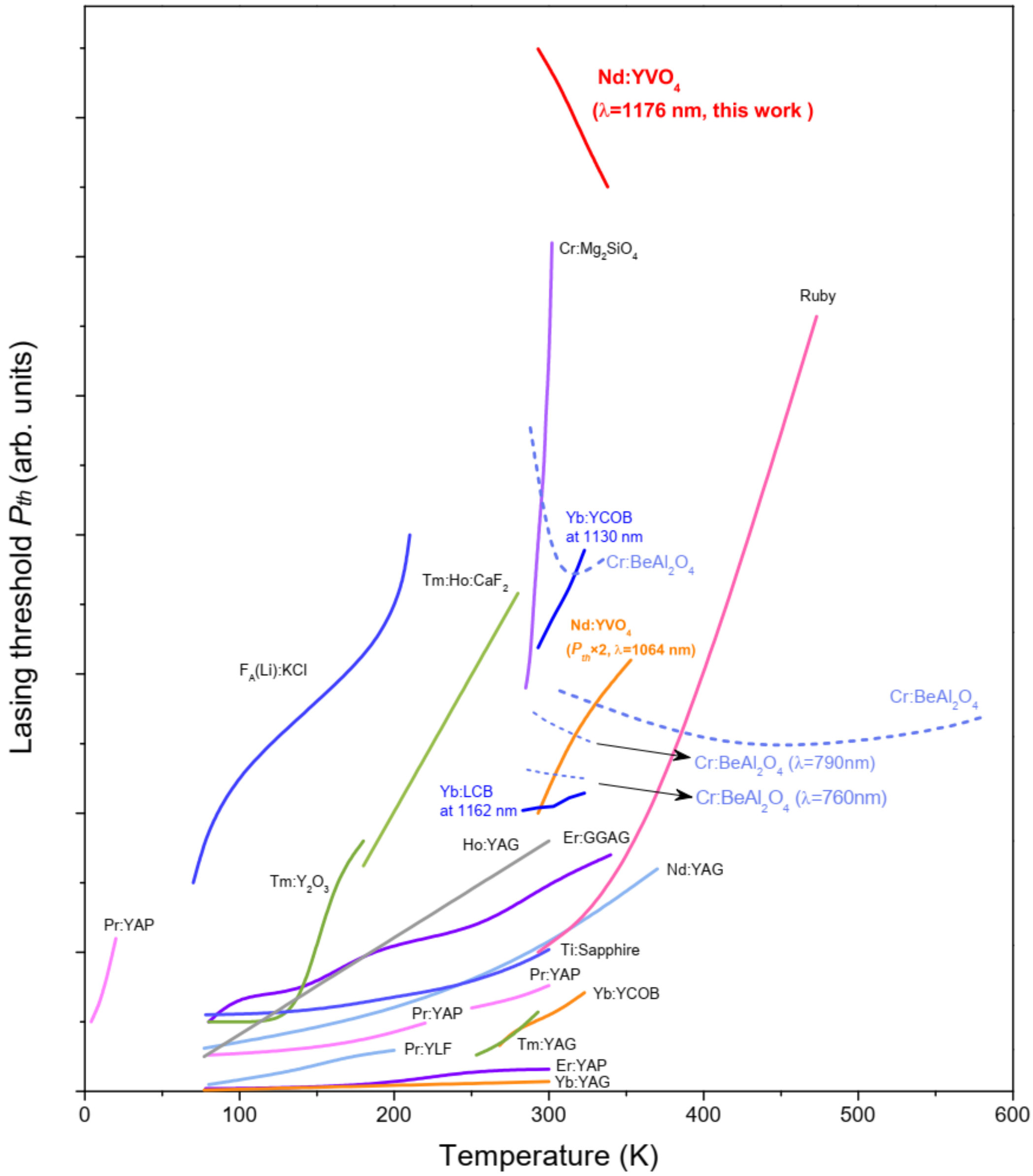

Figure S1. A summary of temperature dependent lasing threshold in solid-state lasers. All solid-state lasers prefer to low temperature with reduced lasing threshold and higher slope efficiency, including ruby laser, Ti:sapphire, Yb:YAG, Tm:YAG, Ho:YAG, Er:GGAG, Pr:YAP, Fe:ZnSe, Nd:YVO_4 (λ=1.064 nm), *etc*. In Fig. S1, all of them have a positive slope with $\partial P_{th}/\partial T$>0. This has been a common sense for solid-state vibronic lasers. However, for our vibronic laser, its $\partial P_{th}/\partial T$ is negative, namely high-temperature is favorable for reducing lasing threshold. Such an anomalous behavior can be attributed to a distinctive phonon-pumping mechanism. In addition, our vibronic laser exhibits a relatively high lasing threshold owing to the weak emission intensity of fluorescence satellites.

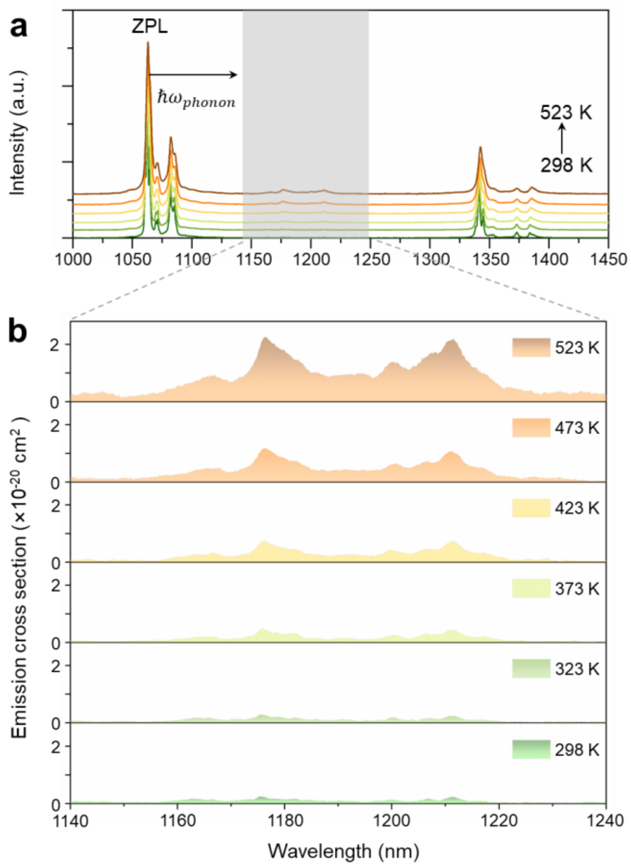

Figure S2. Emission cross sections of Nd:YVO_4 for σ-polarization. **a**, Thermal fluorescence spectra from 298 K to 523 K. **b**, Emission cross sections for σ-polarization in 1140 nm-1240 nm window.

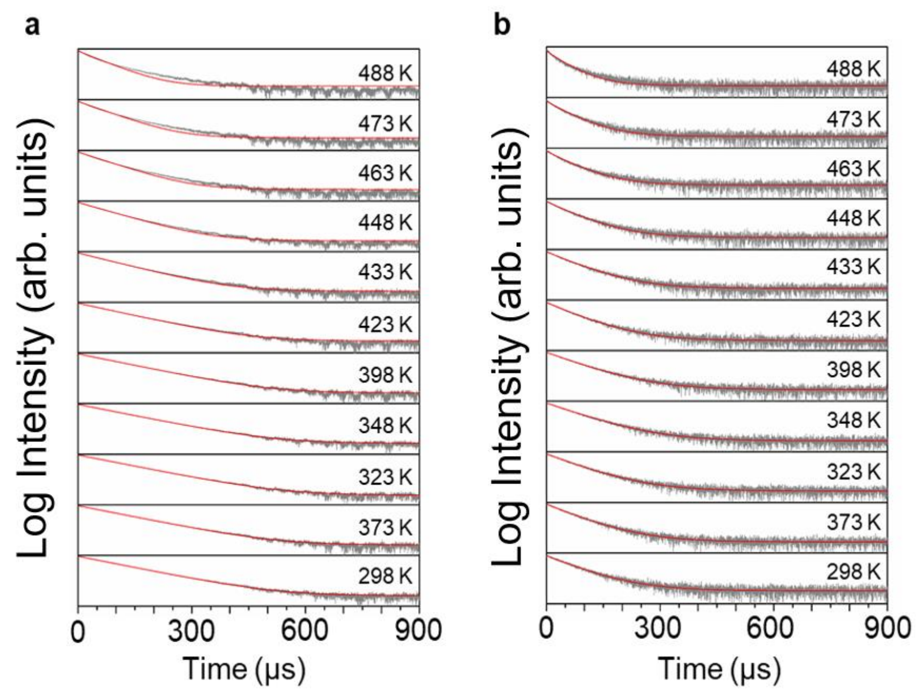

Figure S3. Fluorescence intensity-time decay curves under various temperatures (298 K-488 K). **a**, 1064 nm. **b**, 1176 nm.

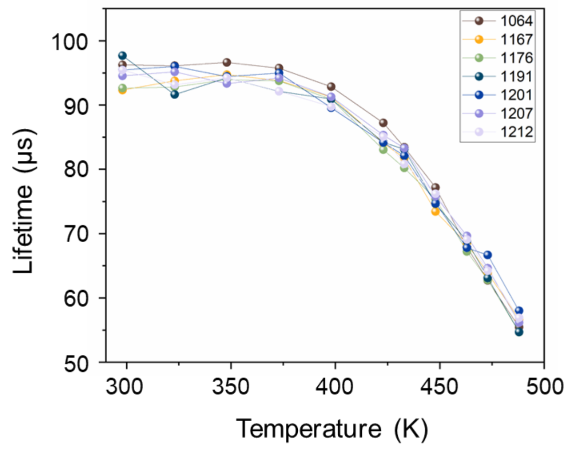

Figure S4. The lifetimes of phonon-triggered emissions at 1167 nm, 1176 nm, 1191 nm, 1201 nm, 1207 nm, 1212 nm under different temperatures.

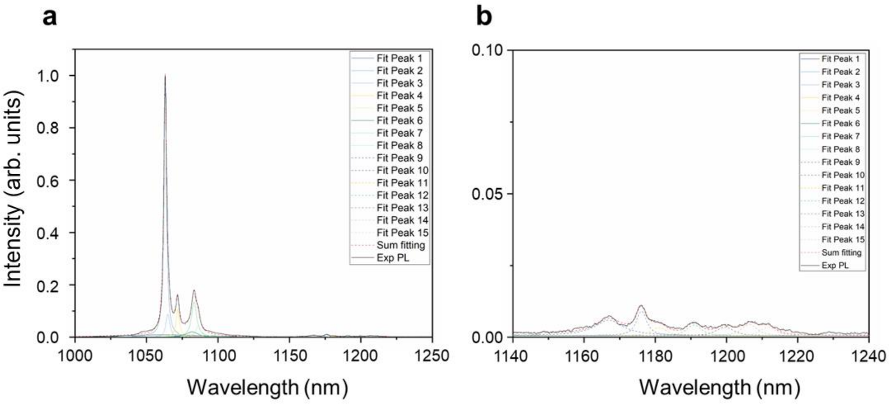

Figure S5. Fitting peaks for π-polarization fluorescence spectra at 523 K. **a**, original graph. **b**, enlarged graph in the phonon-triggered region. The solid lines within 1040 nm-1100 nm represent zero-phonon transitions from the manifolds $^4F_{3/2}$ to $^4I_{11/2}$. The dashed lines around 1150 nm-1210 nm represent phonon-triggered transitions. The calculated *S* factor is 0.013.

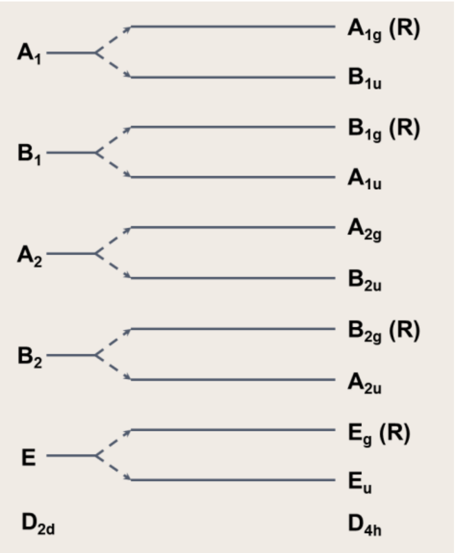

Figure S6. Correlation chart for symmetry species of the D_{2d} group (left) and the D_{4h} group (right)[1].

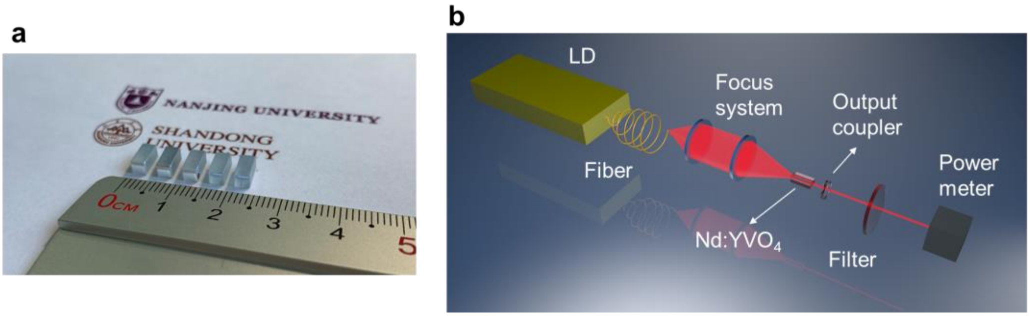

Figure S7. Nd:YVO_4 laser crystals and laser experimental setup. **a**, Photographs of five a-cut Nd^{3+}-doped YVO_4 crystals with dimensions of 7.6×3×3 mm^3. These crystals were utilized in laser experiments for statistical results. **b**, Experimental setup of the Nd:YVO_4 photon-phonon collaboratively pumped laser.

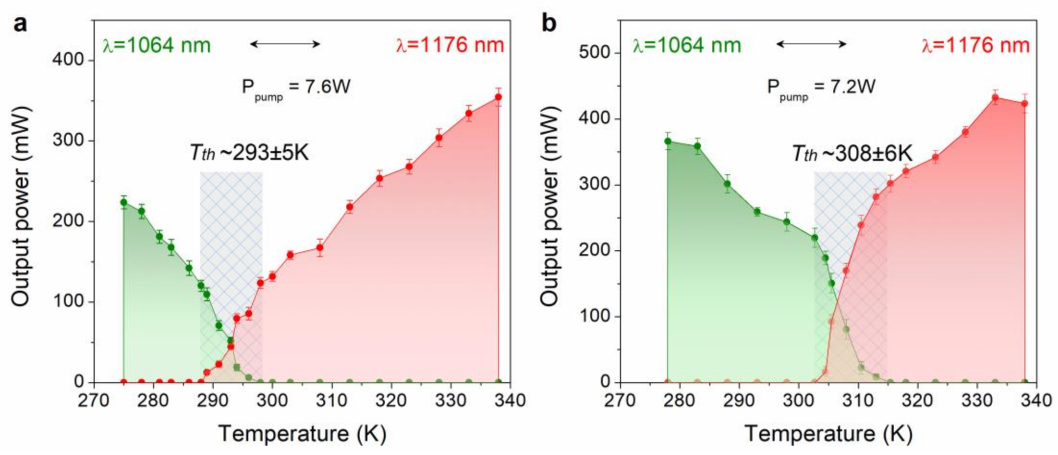

Figure S8. Phase diagram of 1064 and 1176 nm lasing under different pump powers. **a**, P_{pump}=7.6W, **b**, P_{pump}=7.2W. Experimental data are presented as mean values +/- SD with five samples.

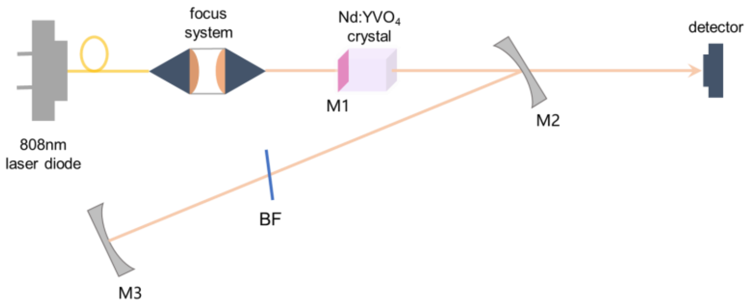

Figure S9. Tunable phonon-pumped laser in one-phonon process.M1 is coated high-reflection (HR, R>99.9%) at 1176 nm, high-transmission (HT, T>99.5%) at 1064 nm and 1342 nm, and HT (T>90%) at 808 nm, 914 nm and 1085 nm. The front surfaces of M2 and M3 are coated with HR at 1176 nm, HT at 914 nm, 1064 nm 1083 nm and 1342 nm. The end surfaces of M2 and M3 are coated with anti-reflection (AR) at 914 nm, 1064 nm 1083 nm and 1342 nm. A thin MgF_2 BF (d=1 mm) is inserted along the Brewster's angle

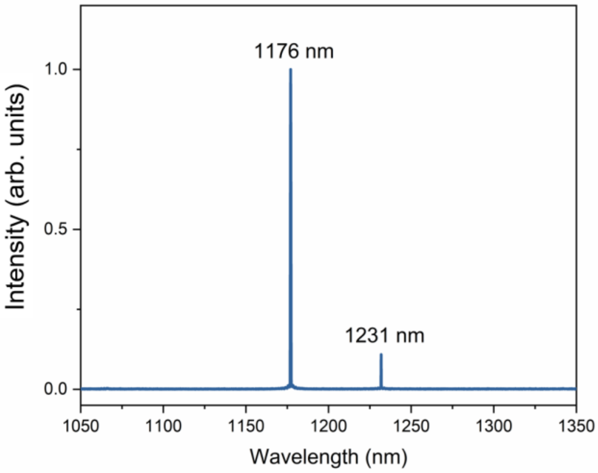

Figure S10. Two-phonon pumped 1231 nm laser in Nd:YVO_4 laser.

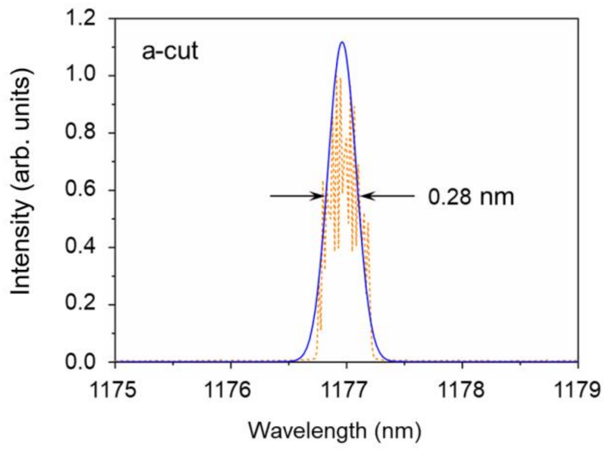

Figure S11. Full width at half maxima (FWHM) of a-cut Nd:YVO_4 laser at 1176 nm. The orange line is experimental laser spectrum and blue line is fitting curve. The resolution of the spectrometer for spectral measurement is 0.02 nm.

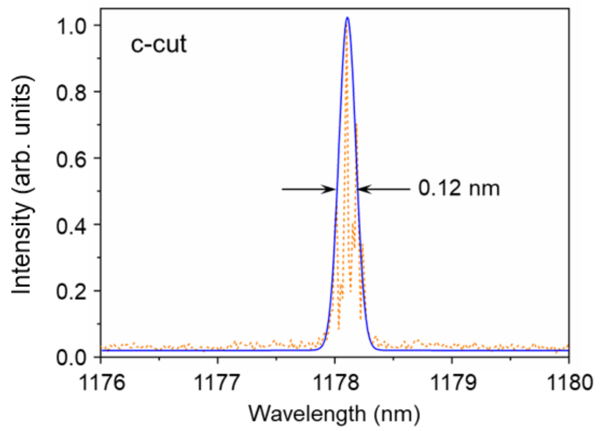

Figure S12. Full width at half maxima (FWHM) of c-cut Nd:YVO_4 laser at 1178 nm. The orange line is experimental laser spectrum and blue line is fitting curve. The resolution of the spectrometer for spectral measurement is 0.02 nm.

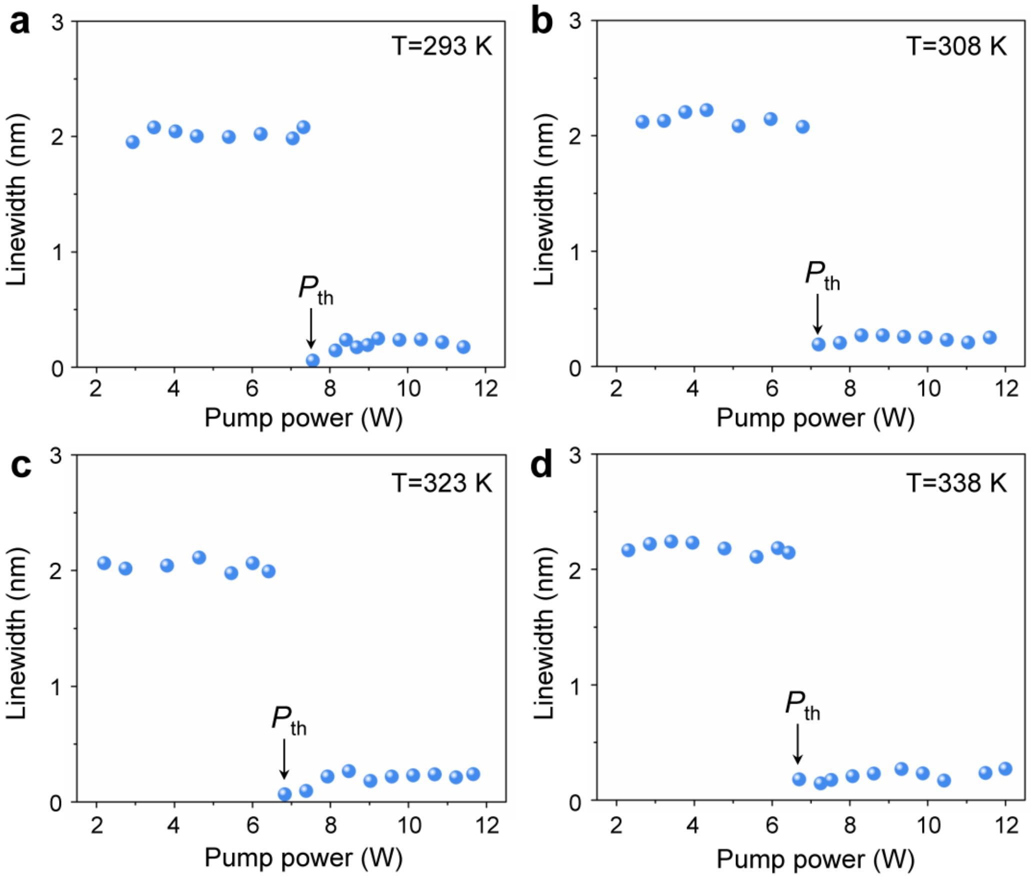

Figure S13. Pump power dependence of the emission linewidths of photon-phonon collaboratively pumped fluorescence (laser) at various cooling temperatures. **a**, 293 K, **b**, 308 K, **c**, 323 K, **d**, 338 K. The linewidths suddenly become narrower when the pump power reaches the threshold at various cooling temperature, which also provides a good evidence for the occurrence of photon-phonon collaboratively pumped lasing within Nd:YVO_4 crystal[2].The increase of laser linewidth can be attributed to multimode laser oscillation.

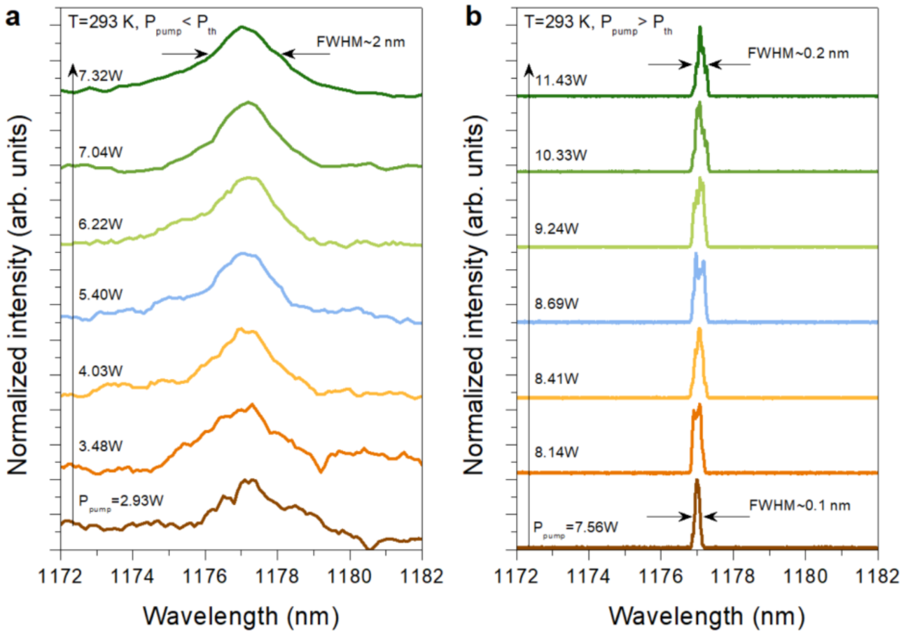

Figure S14. The pump dependent fluorescence and laser spectra under normalized condition, T=293 K. **a**, $P_{pump}<P_{th}$, **b**, $P_{pump}>P_{th}$. The resolution of the spectrometer for spectral measurement is 0.05 nm. With the increasing pump power, there are some multi-mode laser operations.

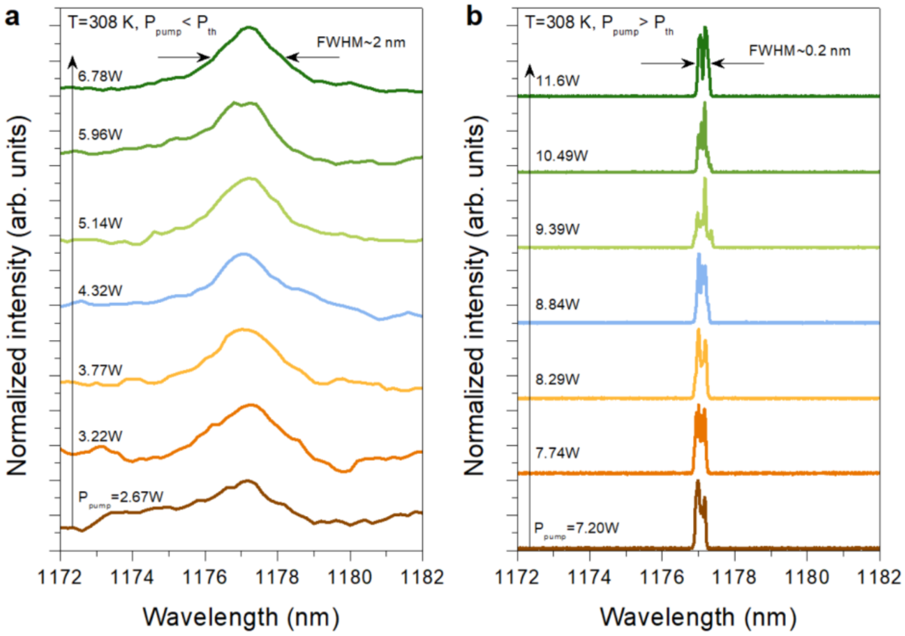

Figure S15. The pump dependent fluorescence and laser spectra under normalized condition, T=308 K. **a**, $P_{pump}<P_{th}$, **b**, $P_{pump}>P_{th}$.

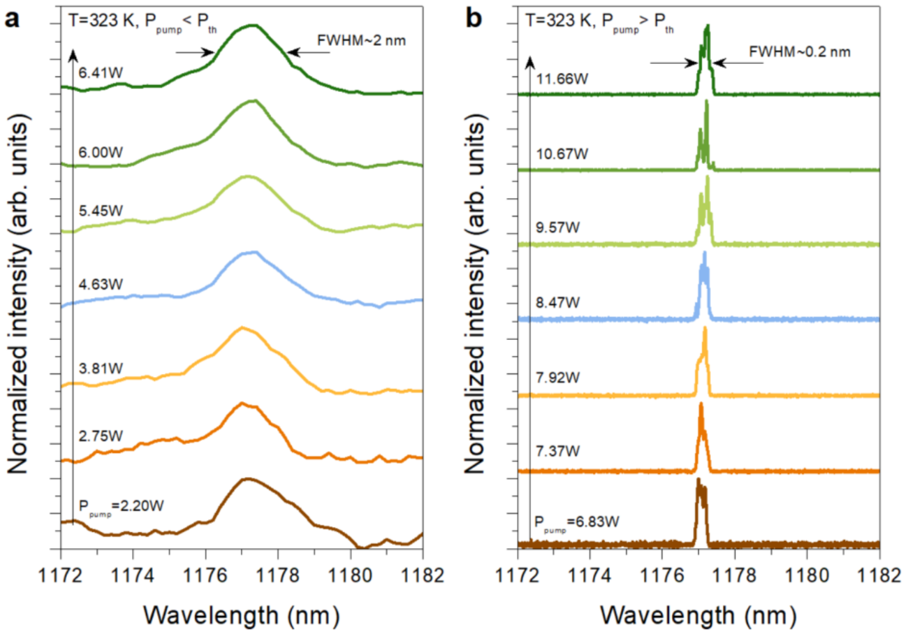

Figure S16. The pump dependent fluorescence and laser spectra under normalized condition, T=323 K. **a**, $P_{pump}<P_{th}$, **b**, $P_{pump}>P_{th}$.

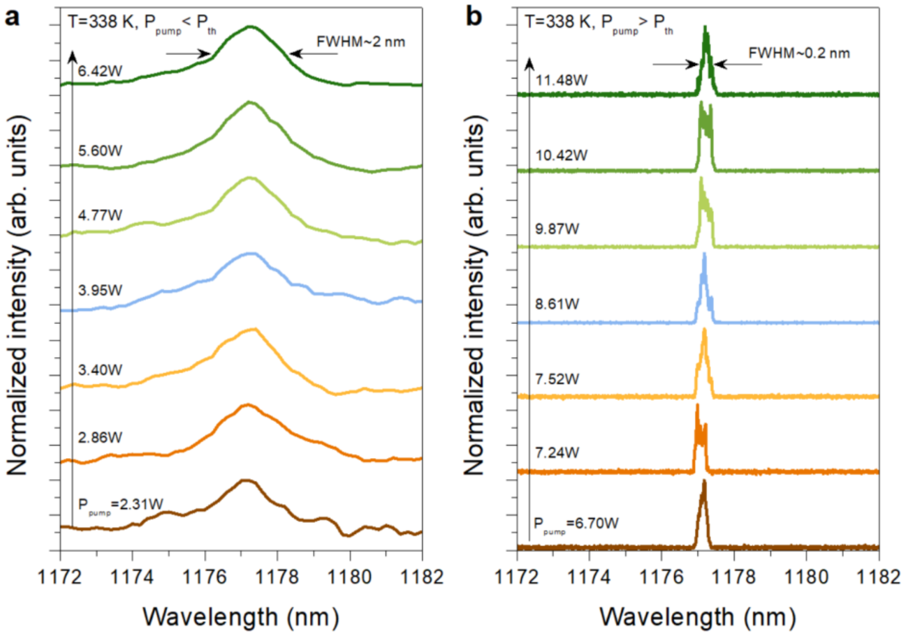

Figure S17. The pump dependent fluorescence and laser spectra under normalized condition, T=338 K. **a**, $P_{pump}<P_{th}$, **b**, $P_{pump}>P_{th}$.

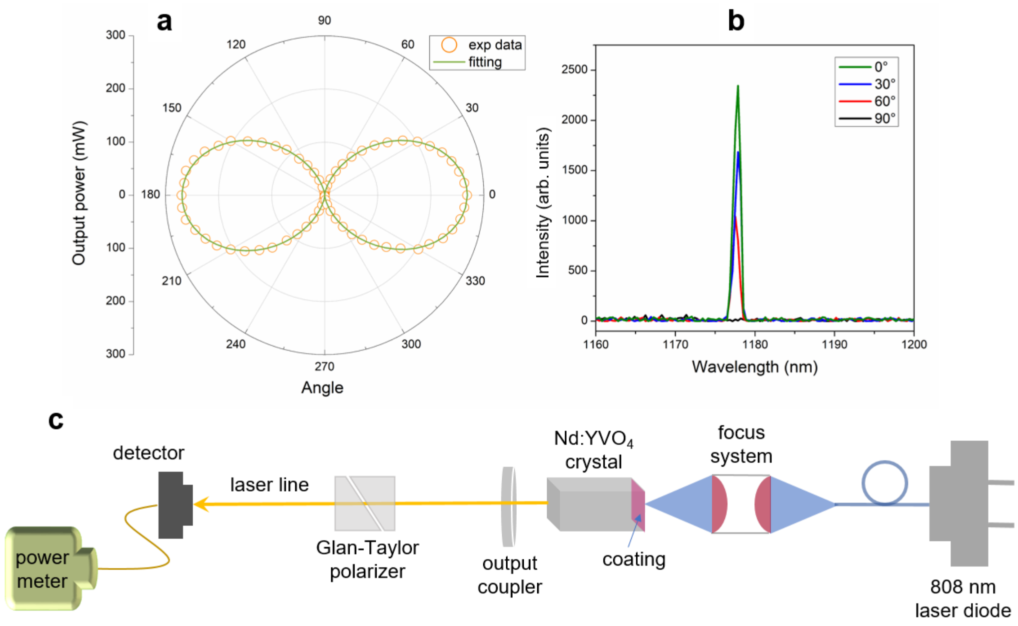

Figure S18. Polarization measurement of Nd:YVO_4 laser. **a**, Polarization dependence of 1176 nm laser. **b**, Laser spectra for various polarization angles. **c**, Experimental setup of the Nd:YVO_4 photon-phonon collaboratively pumped laser.

The non-normalized polarization data was given in the Fig. S18a. We can see that the maximum and minimum laser powers after Glan-Taylor polarizer are 268.3 and 0.036 mW, respectively. So, the degree of polarization (ρ) is 99.97% by the formula of $\rho = \frac{I_{//}-I_{\perp}}{I_{//}+I_{\perp}}$, where $I_{//}$ is the intensity of light parallel to the polarizer, and $I_{\perp}$ is the intensity of light perpendicular to the polarizer[3]. The spectral data was plotted in Fig. S18b. This result indicates our laser is a near-perfect linear-polarized light.

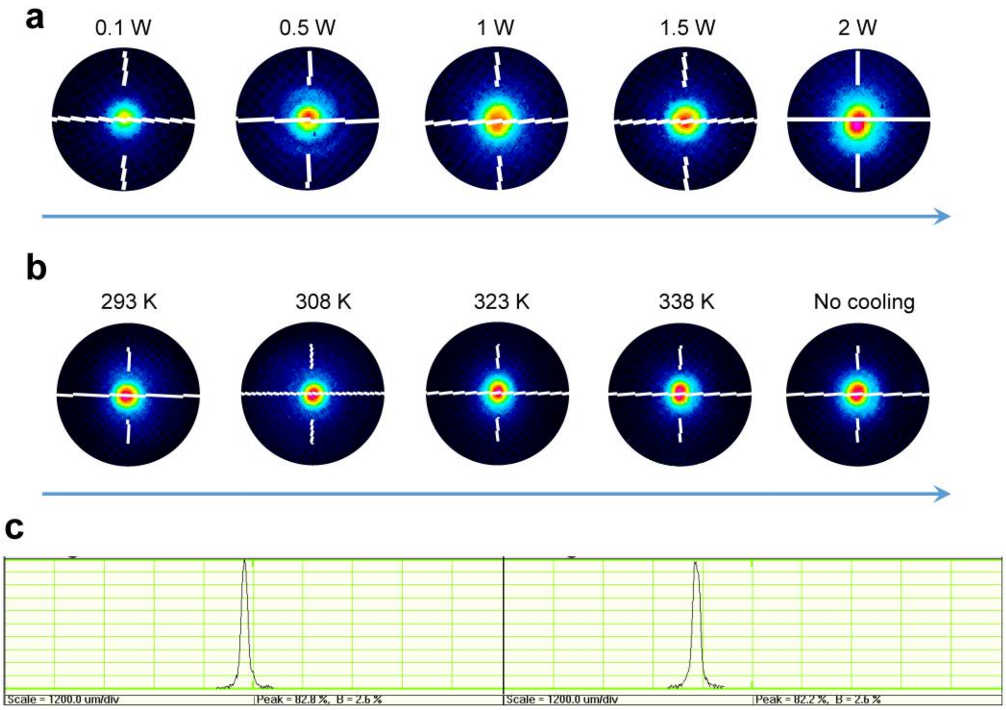

Figure S19. Output beam profiles of Nd:YVO_4 laser. **a**, Output beam profiles of 1176 nm laser with the improved output power. The cooling temperature maintains at 338 K. **b**, Output beam profiles of 1176 nm laser with the improved cooling temperature. The pump power maintains 11 W. **c**, The corresponding one-dimensional intensity profiles of 1.5 W laser without cooling.

As shown in Fig. S19, nearly circular symmetric laser beams are always observed for the various values of output power and cooling temperature. The corresponding one-dimensional intensity profiles are well fitted with the Gaussian distribution, *i.e.*, a typical feature of the laser.

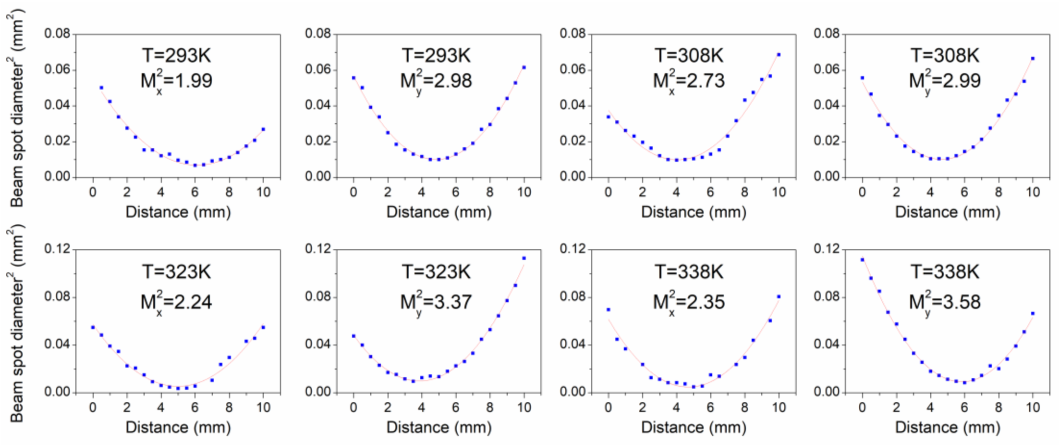

Figure S20. Beam spot radius as a function of distance measured at a crystal temperature of 20-65 °C. R_{oc}= 50 mm. The crystal dimension is 3×3×7.6 mm^3. The beam quality at 293, 308, 323, 338 K were calculated. All beam qualities are measured while the output power maintains 1 W.

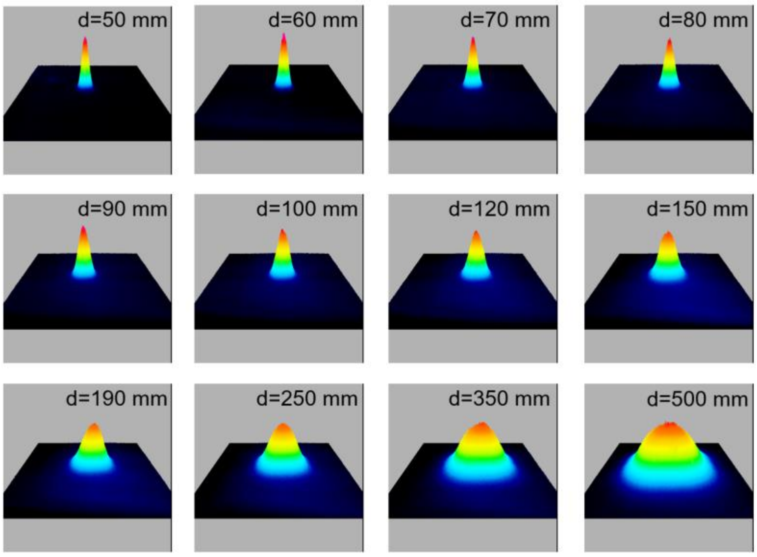

Figure S21. Output beam profiles of 1176 nm laser at 293 K. The output power maintains 1 W. Cavity length (R = 50 mm). *d* represents the distance between output coupler and CCD detector. The crystal dimension is 3×3×7.6 mm^3.

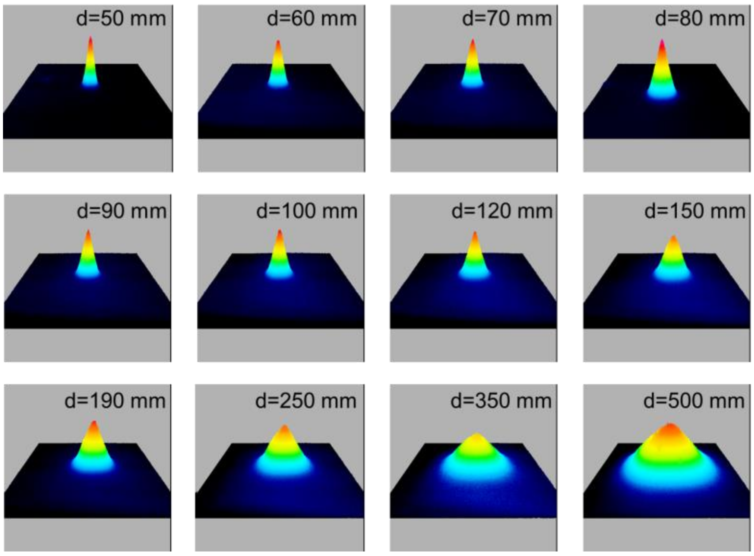

Figure S22. Output beam profiles of 1176 nm laser at 308 K. The output power maintains 1 W. Cavity length (R = 50 mm). *d* represents the distance between output coupler and CCD detector. The crystal dimension is 3×3×7.6 mm^3.

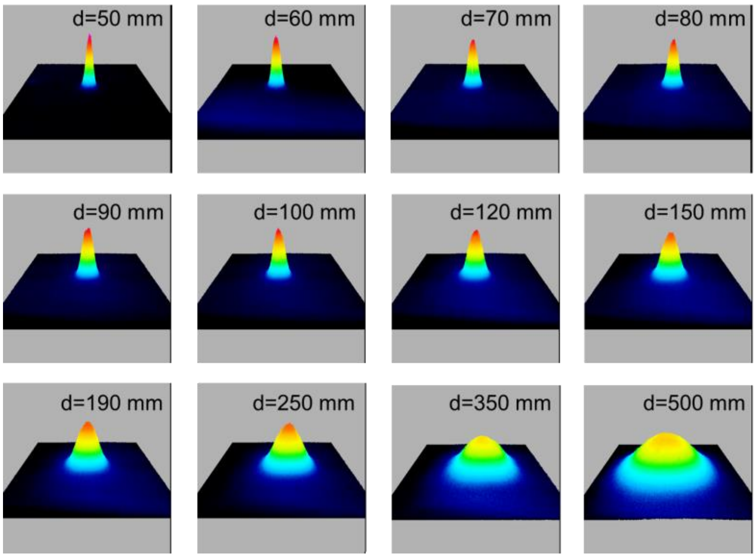

Figure S23. Output beam profiles of 1176 nm laser at 323 K. The output power maintains 1 W. Cavity length (R = 50 mm). *d* represents the distance between output coupler and CCD detector. The crystal dimension is 3×3×7.6 mm^3.

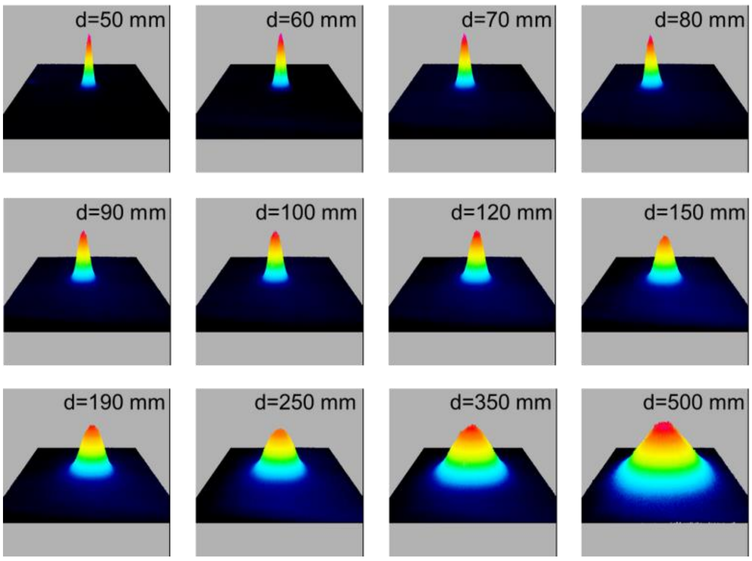

Figure S24. Output beam profiles of 1176 nm laser at 338 K. The output power maintains 1 W. Cavity length (R = 50 mm). *d* represents the distance between output coupler and CCD detector. The crystal dimension is 3×3×7.6 mm^3.

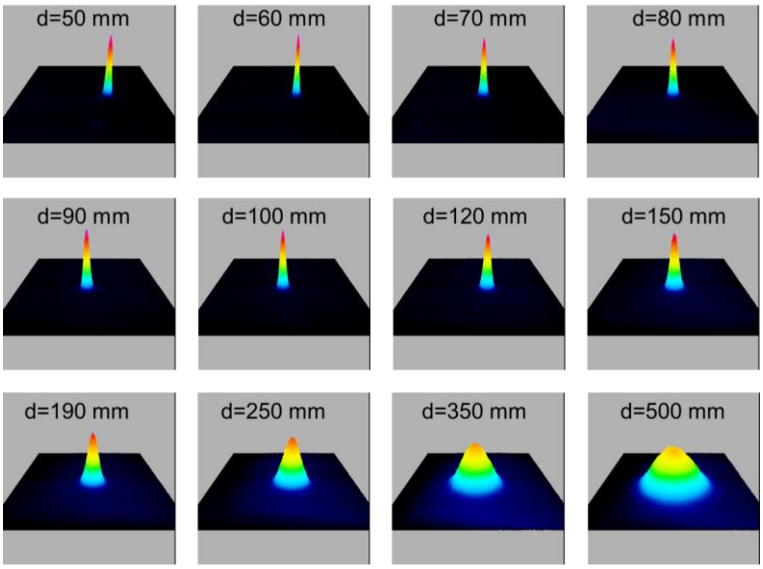

Figure S25. Output beam profiles of 1176 nm laser at 338 K. The output power maintains 1 W. Cavity length (R = 100 mm). *d* represents the distance between output coupler and CCD detector. The crystal dimension is 3×3×7.6 mm^3.

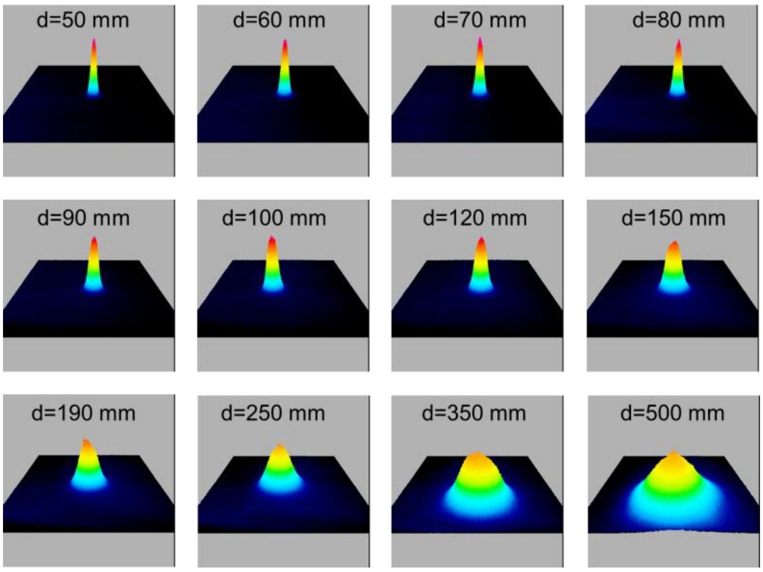

Figure S26. Output beam profiles of 1176 nm laser at 338 K. The output power maintains 1 W. Cavity length (R = 50 mm). *d* represents the distance between output coupler and CCD detector. The crystal dimension is 2×2×7.4 mm^3.

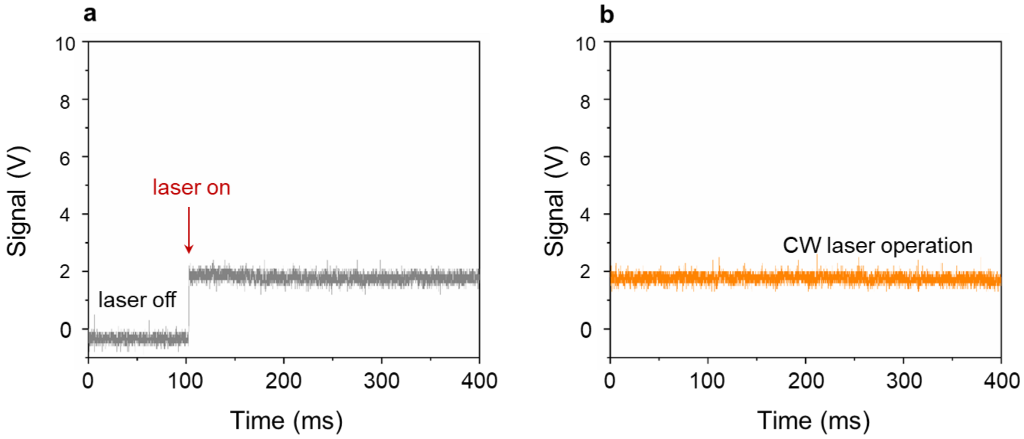

Figure S27. Temporal characteristics of Nd:YVO_4 laser at 1176 nm. **a**. Typical oscilloscope trace of 1176 nm laser emission. **b**. Stable CW laser operation.

As shown in Fig. S27, there is no laser pulse in the oscilloscope trace, indicating that the relaxation oscillation is well suppressed. Therefore, our photon-phonon collaboratively pumped laser is a continuous wave (CW) laser. This case is consistent with the previous CW laser[4].

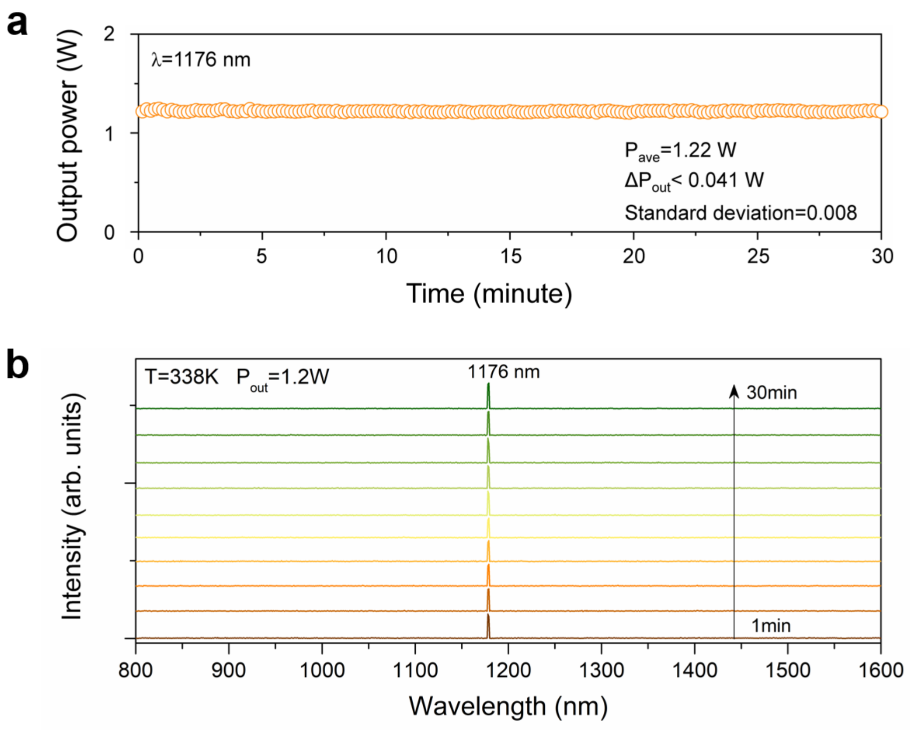

Figure S28. Stability of Nd:YVO_4 laser at 1176 nm. **a**. The power stability measured at an output power of 1.2 W. **b**. The wavelength stability measured at an output power of 1.2 W.

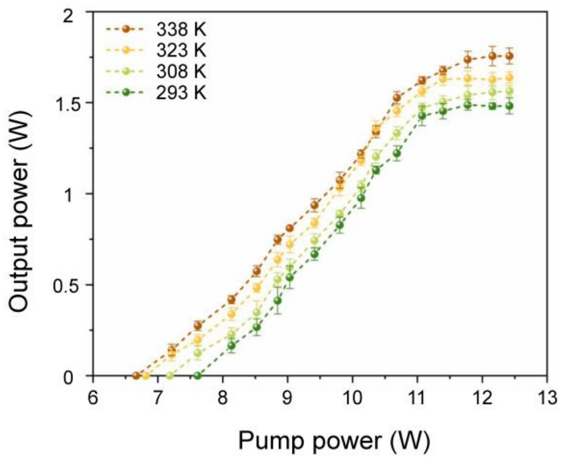

Figure S29. Photon-phonon collaboratively pumped laser performances at the wavelength of 1176 nm with different cooling temperature. Experimental data are presented as mean values +/- SD with five crystal samples. The employed output coupler has the transmittance of 1% and a curvature radius of 50 mm.

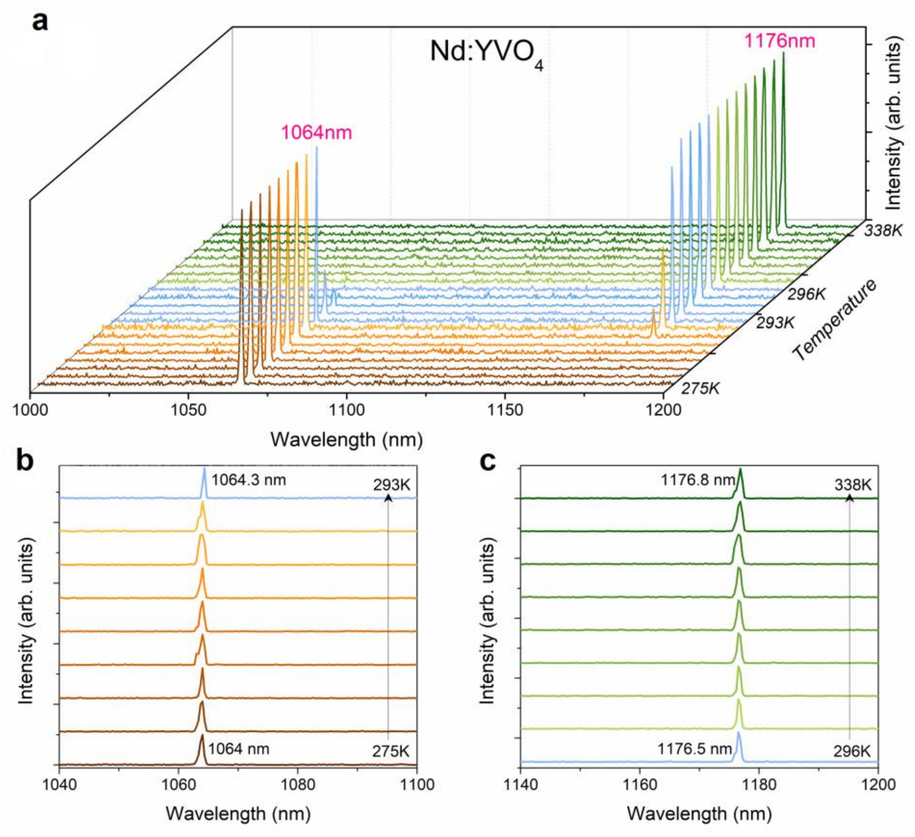

Figure S30. The phonon-pumped laser wavelengths at different temperatures. **a**, 1064 nm and 1176 nm. **b**, 1064 nm, **c**, 1176 nm.

The phonon-pumped laser wavelength is dependent on the temperature. We measured the laser wavelength under different temperatures. As shown in Fig. S30a, there are two lasing wavelengths, 1064 nm at low temperature and 1176 nm at high temperature. The transition temperature locates around 293-296 K. We found that there is a wavelength shift from 1176.5 to 1176.8 nm with increasing temperature from 296 to 338 K. This shift can be exactly explained by our proposed laser theory. According to reference[5] [see Fig. S35b], the ZPL wavelength shifts from 1064 to 1064.25 nm with increasing temperature from 20 to 65 °C. This is consistent with our results in Fig. S30b. Moreover, the phonon wavenumber of A_{1g} mode shifts from 890.86 cm^{-1} to 890.46 cm^{-1} with increasing temperature from 296 to 338 K. Accordingly, the phonon-pumped lasing wavelength slightly shifts from 1175.47 to 1175.66 nm. This agrees well with our experimental results in Fig. S30c if considering measurement errors.

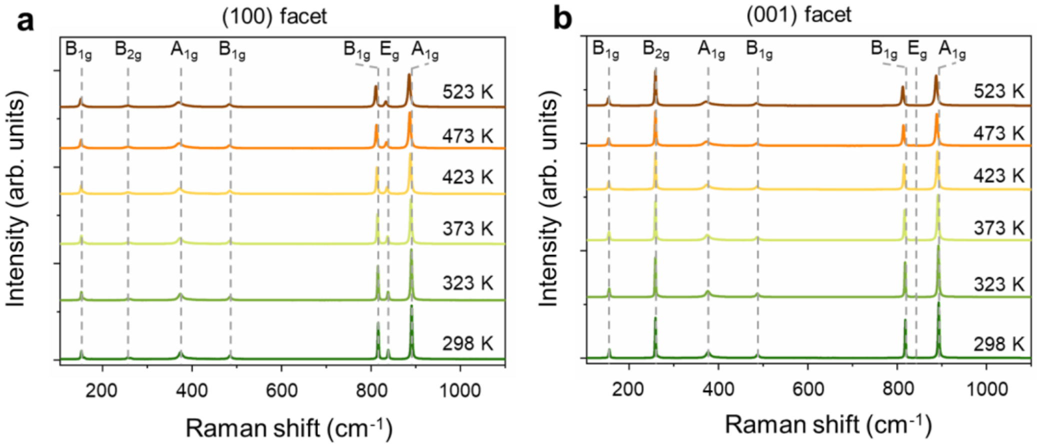

Figure S31. Temperature dependent Raman spectrum of the Nd:YVO_4 crystal for two different crystal orientations. **a**, (100) facet, **b**, (001) facet. The incident lasers are parallel to a-axis and c-axis, respectively.

Table S1. A summary of temperature-dependent solid-state laser performances

Laser crystal	Temperature (K)	Threshold (a.u.)	Slope efficiency	References
Ruby<br>λ ~ 0.694 μm	293 K	700 watts/cm^3	N/A	Journal of Applied Physics (1962), 33, 2333-2335.
	363 K	1100 watts/cm^3	N/A	
	473 K	3900 watts/cm^3	N/A	
F_A(Li) center in KCl<br>λ ~ 2.7 μm	70 K	1.5 (a.u.)	N/A	Solid State Communications (1965), 3, 61-63.
	90 K	2.3 (a.u.)	N/A	
	200 K	3.2 (a.u.)	N/A	
	210 K	4.0 (a.u.)	N/A	
Co^{2+}:MgF_2<br>λ ~ 1.94 μm	80 K	6 mJ	15.9%	IEEE Journal of Quantum Electronics (1985), 21, 1582-1595.
	225 K	8 mJ	12.7%	
Ti:Sapphire<br>λ ~ 0.75 μm	78 K	0.55 W	24%	Laser Physics (2018), 28, 085801.
	120 K	0.56 W	N/A	
	160 K	0.6 W	N/A	
	200 K	0.68 W	N/A	
	240 K	0.75 W	N/A	
	280 K	0.9 W	N/A	
	300 K	1.02 W	17%	
alexandrite<br>Cr^{3+}:$BeAl_2O_4$<br>λ ~ 0.76 μm	286K	3.42W	N/A	Opt. Express (2023), 31, 9790-9799.
	296K	3.26W	N/A	
	306K	3.17W	N/A	
	316K	3.06W	N/A	
	326K	2.98W	N/A	
alexandrite<br>Cr^{3+}:$BeAl_2O_4$<br>λ ~ 0.79 μm	290K	6.16W	N/A	Opt. Express (2023), 31, 9790-9799.
	299K	5.71W	N/A	
	309K	5.35W	N/A	
	318K	5.04W	N/A	
	328K	4.71W	N/A	
alexandrite<br>Cr^{3+}:$BeAl_2O_4$<br>λ ~ 0.75-0.79 μm	307	7.2 J	N/A	Opt. Lett. (1982), 7, 608-610.
	378	5.2 J	N/A	
	445	4.3 J	N/A	
	548	5.2 J	N/A	
	583	6.0 J	N/A	
alexandrite<br>Cr^{3+}:$BeAl_2O_4$<br>λ ~ 0.75-0.79 μm	288	19.8 J	N/A	IEEE J. Quantum Electron. (1980), 16, 1302-1315.
	301	13.6 J	N/A	
	315	12.4 J	N/A	
	337	13.6 J	N/A	
Cr^{4+}:Mg_2SiO_4<br>λ ~ 1.3 μm	285 K	2.9 W	N/A	IEEE Journal of Quantum Electronics (1998), 34, 1996-2005.
	288 K	3.1 W	N/A	
	290 K	3.5 W	N/A	
	294 K	4.1 W	N/A	
	297 K	4.5 W	N/A	
	298 K	4.9 W	N/A	
	300 K	5.2 W	N/A	
	302 K	6.1 W	N/A	
Cr^{4+}:YAG<br>λ ~ 1.45 μm	276 K	1.08 W	42.1%	Journal of the Optical Society of America B (1995), 12, 930-937.
	293 K	1.215 W	34.2%	
Cr^{2+}:ZnSe<br>λ ~ 2.2-2.4 μm	78 K	6 mJ	6.7%	Proc. SPIE (2019), 110330Y.
	180 K	6.5 mJ	6%	
	300 K	9 mJ	5.7%	
	380 K	15 mJ	3.4%	

Fe^{2+}:ZnSe λ ~ 3.98 μm	19 K	1.2 μJ	3.2%	Optics Letters (1999), 24, 1720-1722.
	70 K	3.0 μJ	1.7%	
Fe^{2+}:ZnSe λ ~ 4.54 μm	150 K	24.5 μJ	8.2%	Optics Letters (1999), 24, 1720-1722.
	170 K	46.3 μJ	5.2%	
Fe^{2+}:ZnSe λ ~ 4.1 μm	85 K	15 mJ	43%	IEEE Journal of Quantum Electronics, (2006), 42, 907-917.
	186 K	~ 35 mJ	~37%	
	220 K	~ 60 mJ	~28%	
Nd:YAG λ ~ 0.946 μm	243 K	50 J	10%	Applied Physics B (1991), 53, 6-10.
	248 K	50 J	10%	
	253 K	52 J	9.5%	
	263 K	57 J	9%	
	273 K	62 J	7%	
	283 K	70 J	6%	
	298 K	74 J	2%	
YAG/Nd:YAG/YAG composite rod λ ~ 0.946 μm	293 K	1.86 W	29%	Applied Optics (1998), 37, 5713-5719.
	298 K	1.92 W	27.8%	
	303 K	1.92 W	26.8%	
	308 K	2.1 W	23.5%	
	313 K	2.08 W	22%	
	318 K	2.13 W	20%	
	323 K	2.12 W	19%	
Nd:YAG λ ~ 1.06 μm	30 K	17.5 J	10%	Optics Communications (1989), 73, 62-66.
	77 K	14 J	NA	
	100 K	14.5 J	16.5%	
	150 K	16 J	NA	
	200 K	20 J	22%	
	250 K	21.5 J	NA	
	300 K	23 J	23%	
Nd:YAG λ ~ 1.0612 μm	77 K	0.31 (a.u.)	N/A	Journal of the Optical Society of America (1966), 56, 1409-1410.
	195 K	0.54 (a.u.)	N/A	
	295 K	1 (a.u.)	N/A	
	370 K	1.6 (a.u.)	N/A	
Cr:Nd:GSGG λ ~ 1 μm	213 K	4.88 J	N/A	IEEE Journal of Quantum Electronics (2003), 39, 741-748.
	233 K	5.02 J	N/A	
	253 K	5.17 J	N/A	
	273 K	5.35 J	N/A	
	293 K	5.55 J	N/A	
	313 K	5.78 J	N/A	
	333 K	6.04 J	N/A	
	353 K	6.34 J	N/A	
Nd:YVO_4 λ ~ 0.914 μm	278 K	NA	high	Jurnal Teknologi (Sciences & Engineering) (2016), 78, 149-154.
	333 K	NA	low	
Nd:YVO_4 λ ~ 1.064 μm	273 K	N/A	high	Appl. Opt. (1993), 32, 2085-2086
	373 K	N/A	low	
Nd:YVO_4 λ ~ 1.06 μm	293 K	1 W	N/A	Journal of the Optical Society of America B (2011), 28, 972-976.
	318 K	1.32 W	N/A	
	353 K	1.55 W	N/A	
Nd:YVO_4 λ ~ 1.342 μm	293 K	NA	good	Optics Communications (1999), 164, 191–197
	313 K	NA	poor	
Nd:Y_2O_3 λ ~ 1.073 μm	77 K	0.34 (a.u.)	N/A	Journal of the Optical Society of America (1966), 56, 1409-1410.
	195 K	0.64 (a.u.)	N/A	
	295 K	1 (a.u.)	N/A	
	370 K	1.78 (a.u.)	N/A	
Nd:$LaSc_3(BO_3)_4$ λ ~ 1.062 μm	77 K	~ 38 mW	44%	Applied Physics B Lasers and Optics, (1994), 58, 381-388.
	300 K	~ 40 mW	38%	

Yb:YAG<br>λ ~ 1.03 µm	77 K	~ 9 mW	67%	Optics Letters,<br>(1991), 16, 1089-1091.
	300 K	71 mW	~43%	
Yb:GGAG<br>λ ~ 1.026 µm	100 K	2.5 mJ	36%	Proc. SPIE, High-Power, High-Energy, and High-Intensity Laser Technology III, (2017), 1023812
	200 K	4.3 mJ	31%	
	300 K	12.6 mJ	11%	
Yb:$Y_{0.77}Gd_{0.09}Ca_4O(BO_3)_3$<br>λ ~ 1.084 µm	268 K	0.33 W	60%	Optics Communication<br>(2018), 427, 244-249.
	278 K	~ 0.45 W	58%	
	303 K	~ 0.55 W	55%	
	323 K	0.71 W	51%	
Yb: $YCa_4O(BO_3)_3$<br>λ ~ 1.023 µm	278 K	3.7 W	24%	Optics & Laser Technology<br>(2018), 108, 360-363.
	323 K	6.75 W	20%	
Yb: $YCa_4O(BO_3)_3$<br>λ ~ 1.13 µm	293K	3.19W	N/A	Nature Physics (2022), 18, 1312-1316.
	303K	3.41W	N/A	
	313K	3.62W	N/A	
	323K	3.89W	N/A	
Yb: $La_2CaB_{10}O_{19}$<br>λ ~ 1.162 µm	283K	1.46W	8.4%	Light: Science & Applications<br>(2023) 12:203
	293K	1.59W	8.3%	
	303K	1.66W	8.2%	
	313K	2.07W	8.1%	
	323K	2.30W	7.4%	
Tm:YAG<br>λ ~ 2.013 µm	253 K	0.26 W	44%	Optics Express (1999), 4, 12-18.
	273 K	0.36 W	34%	
	293 K	0.57 W	27%	
Tm:Y_2O_3<br>λ ~ 1.932 µm	80 K	0.5 W	32.2%	Applied Physics B (2020), 126, 44
	100 K	0.5 W	29.7%	
	120 K	0.5 W	26.5%	
	140 K	0.6 W	22.6%	
	160 K	1.5 W	21.6%	
	180 K	1.8 W	18.7%	
Tm:$GdVO_4$<br>λ ~ 1.84 µm	78 K	1 W	44%	Optica High-brightness Sources and Light-driven Interactions Congress 2022, Technical Digest Series (2022), paper JW5A.14.
	200 K	2.1 W	12%	
Tm:Lu:CaF_2<br>λ ~ 1.86 µm	78 K	1 W	65.5%	Journal of Luminescence<br>(2023), 255, 119563
	200 K	1.2 W	55.9%	
	300 K	1.2 W	38%	
Ho:YAG<br>λ ~ 2.097 µm	77 K	0.25 W	43%	Proceedings of SPIE (2009), 71931H.
	300 K	1.8 W	28%	
Ho:YLF<br>λ ~ 2.055 µm	77 K	45 mW	28%	Applied Physics B (1994), 58, 69-71.
	210 K	80 mW	18%	
Tm:Ho:CaF_2<br>λ ~ 2.1 µm	180 K	1.62 W	15.7%	Advanced Solid State Lasers, (2014), paper ATh2A.14.
	280 K	3.58 W	8.6%	
Tm:Ho:YLF<br>λ ~ 2.065 µm	100 K	~ 230 mW	N/A	Chinese Optics Letters (2003), 1, 281-282.
	170 K	~ 320 mW	N/A	
	200 K	~ 460 mW	N/A	
	220 K	~ 750 mW	N/A	
Pr:YLF<br>λ ~ 0.607 µm	80 K	49 mW	46%	Laser Phys. Lett. (2015), 12, 095801
	110 K	100 mW	44%	
	140 K	160 mW	42%	
	170 K	250 mW	40%	
	200 K	295 mW	36%	
Pr:YAP<br>λ ~ 0.493 µm	4 K	500 mW	26%	Applied Physics B<br>(2021) 127, 2
	8 K	600 mW	N/A	
	10 K	650 mW	N/A	
	15 K	850 mW	N/A	

	20 K	1100 mW	N/A	
Pr:YAP λ ~ 0.622 µm	80 K	260 mW	13.5%	Laser Phys. Lett. (2014), 11, 105801
	130 K	290 mW	10.4%	
	180 K	360 mW	4.9%	
	220 K	490 mW	2.6%	
Pr:YAP λ ~ 0.662 µm	250 K	600 mW	13.4%	Laser Phys. Lett. (2014), 11, 105801
	280 K	670 mW	10.5%	
	300 K	760 mW	7.9%	
Pr:YAP λ ~ 0.747 µm	80 K	330 mW	55.1%	Laser Phys. Lett. (2014), 11, 105801
	130 K	360 mW	50.9%	
Er:CaF_2 λ ~ 2.7 µm	80 K	24 mW	2.3%	Proc. SPIE, Solid State Lasers XXIV: Technology and Devices, (2015), 93421S
	180 K	135 mW	2%	
	280 K	183 mW	1.3%	
Er:YLF λ ~ 2.8 µm	80 K	2 mJ	22.8%	Proc. SPIE, Solid State Lasers XXVIII: Technology and Devices, (2019), 108961L
	100 K	2 mJ	20.7%	
	300 K	5 mJ	16.4%	
Er:GGAG λ ~ 1.6 µm	80 K	0.5 W	54%	Opt. Mater. Express (2020), 10, 1249-1254
	100 K	0.7 W	52%	
	140 K	0.7 W	52%	
	180 K	1 W	51%	
	220 K	1.1 W	45%	
	260 K	1.2 W	41%	
	300 K	1.5 W	37%	
	340 K	1.7 W	29%	
Er:GGAG λ ~ 2.92 µm	80 K	16 mJ	9.9%	Proc. SPIE 11259, Solid State Lasers XXIX: Technology and Devices, (2020), 112591Q
	100 K	15 mJ	8.7%	
	200 K	15 mJ	7.6%	
	300 K	17 mJ	6.4%	
Er:Pr:GGAG λ ~ 2.9 µm	80 K	210 mW	16%	Proceedings Volume 11980, Solid State Lasers XXXI: Technology and Devices; (2022), 119800A
	100 K	250 mW	12%	
	300 K	270 mW	6%	
Er:YAP λ ~ 2.7 µm	78 K	0.02 W	1.27%	Proc. SPIE, Solid State Lasers XXVII: Technology and Devices, (2018), 1051121
	100 K	0.02 W	1.26%	
	150 K	0.03 W	1.07%	
	200 K	0.06 W	0.79%	
	250 K	0.15 W	0.31%	
	300 K	0.16 W	0.14%	

Table S2. Beam quality parameters of 1176 nm laser at different temperatures.

$Nd:YVO_4$ crystal size and cavity length	Water-cooling temperature (K)	M_x^2	M_y^2	Beam spot radius (μm)	Beam divergence (mrad)
3×3×7.6 mm^3 plane-concave (R_{oc} = 50 mm)	293	1.99	2.98	63.36	5.91
	308	2.73	2.99	62.42	6.0
	323	2.24	3.37	60.46	6.19
	338	2.35	3.58	62.58	5.98
3×3×7.6 mm^3 plane-concave (R_{oc} = 100 mm)	338	1.84	1.95	99.65	3.76
2×2×7.4 mm^3 plane-concave (R_{oc} = 50 mm)	338	3.28	2.65	76.65	4.88

Table S3. Summary of phonon-triggered emissions with their coupling phonon mode and ZPLs

ZPL/nm	Phonon-triggered emission (nm)	Frequency (shift /cm^{-1})	Phonon mode/cm^{-1}
1064.1 ($\Gamma_6 \rightarrow \Gamma_7$)	1167.9	835.2	839 (E_g)
1064.1 ($\Gamma_6 \rightarrow \Gamma_7$)	1177.0	901.4	891 (A_{1g})
1072.8 ($\Gamma_7 \rightarrow \Gamma_6$)	1179.2	841.1	839 (E_g)
1072.8 ($\Gamma_7 \rightarrow \Gamma_6$)	1183.8	874.0	891 (A_{1g})
1084.4 ($\Gamma_7 \rightarrow \Gamma_7$)	1191.2	827.5	816 (B_{1g})
1343.6 ($\Gamma_7 \rightarrow \Gamma_6$)	1201.1	883.0	891 (A_{1g})
1343.6 ($\Gamma_7 \rightarrow \Gamma_6$)	1207.8	836.8	839 (E_g)
1345.4 ($\Gamma_6 \rightarrow \Gamma_6$)	1212.9	812.0	816 (B_{1g})

* Γ_6 and Γ_7 in parentheses are the irreducible representations of electronic states of Nd:YVO_4.

Laser statistics and lifetime

In order to test the repeatability of our laser, we study the laser performances of five Nd:YVO_4 crystals (**Table S4**).

Table S4. Statistical results of Nd:YVO_4 crystals

Wavelength (nm)	Sample	Nd-doping at%	Dimension (mm^3)	Threshold P_{th} (W)	Max P_{out} (W)
1176 (T=293 K)	1#	0.46 %	3*3*7.6	7.56	1.491
	2#	0.46 %	3*3*7.6	7.54	1.52
	3#	0.46 %	3*3*7.6	7.73	1.424
	4#	0.46 %	3*3*7.6	7.56	1.483
	5#	0.46 %	3*3*7.6	7.68	1.467
1176 (T=308 K)	1#	0.46 %	3*3*7.6	7.18	1.558
	2#	0.46 %	3*3*7.6	7.12	1.585
	3#	0.46 %	3*3*7.6	7.3	1.588
	4#	0.46 %	3*3*7.6	7.1	1.524
	5#	0.46 %	3*3*7.6	7.26	1.504
1176 (T=323 K)	1#	0.46 %	3*3*7.6	6.84	1.62
	2#	0.46 %	3*3*7.6	6.73	1.633
	3#	0.46 %	3*3*7.6	6.9	1.616
	4#	0.46 %	3*3*7.6	6.79	1.685
	5#	0.46 %	3*3*7.6	6.86	1.624
1176 (T=338 K)	1#	0.46 %	3*3*7.6	6.68	1.701
	2#	0.46 %	3*3*7.6	6.55	1.743
	3#	0.46 %	3*3*7.6	6.77	1.799
	4#	0.46 %	3*3*7.6	6.61	1.78
	5#	0.46 %	3*3*7.6	6.72	1.758

Section-II: Alternative explanations for Nd:YVO_4 laser

There are also some possible explanations for unprecedented lasing, *e.g.* spontaneous amplification, Raman scattering, modification of fluorescence spectrum by the cavity. However, all of them can be precluded in our photon-phonon collaboratively pumped laser.

First, spontaneous amplification is impossible for our lasing, because there is an obvious laser threshold. In addition, the narrow linewidth can rule out this mechanism.

Second, it is possible to involved phonon into lasing process by Raman scattering, such as self-Raman lasers. However, this is impossible for our reported lasing because there are no excitation sources for the stimulated Raman effect, a type of third-order optical nonlinearity. For example, in the well-known Nd:YVO_4 self-Raman lasers by employing the cascading process of lasing and Raman shifting, the fundamental lasers, *e.g.* at the wavelength of 1064 nm or 1342 nm, should oscillate firstly, and then generate the stimulated Raman shifting effect by the photon-lattice interaction. Therefore, it is necessary to obtain high-intensity fundamental lasers at 1064 nm or 1342 nm laser as the pump sources for Nd:YVO_4 self-Raman laser. Accordingly, the cavity mirrors coating for Nd:YVO_4 self-Raman laser should be high reflection (HR) at 1064 nm or 1342 nm on both surfaces for high-intensity fundamental lasers, and simultaneous partial reflection around 1176 nm or 1500 nm on the output couplers[6,7]. Therefore, there are 1064 and 1176 nm laser simultaneously in the self-Raman laser spectrum (Fig. S32a).

However, in our present work, the photon-phonon collaboratively pumped laser is generated by directly lasing based on the phonon triggered, and there are no Raman scattering processes. In our experimental setup, the cavity mirrors are high-transmittance ($T > 99.5\%$) at 1064 and 1342 nm and high-reflection ($R > 99.9\%$) at 1176 nm. For achieving efficient lasers, the output couplers with partial reflection at 1176 nm are also adopted. Therefore, our lasing is not Raman scattering. In our laser spectrum, there is no 1064 nm laser (Fig. S32b).

Besides, Raman laser doesn't have a temperature threshold and the Raman laser intensity is directly proportional to phonon density of states. However, our 1176 nm laser exhibited an anomalous temperature behavior that increased output power and reduced threshold at high temperatures. There is an obvious temperature threshold T_{th}. This differs from previous YVO_4 Raman laser or Nd:YVO_4 self-Raman laser. In 2012, Wan. et al. reported that there was no obvious change of output power at water temperature of 5, 20, and 40 °C for 1178 nm laser in *c*-Nd:YVO_4 self-Raman laser and *c*-Nd:YVO_4/YVO_4

Raman laser[8]. This is consistent with the result of Zverev[9], who shows that Raman gain coefficient of YVO_4 was not highly sensitive to temperature. In 2015, Ding et al. also reported a similar result in an eye-safe self-Raman Nd:YVO_4 laser at 1525 nm[10], that the maximum output power declined from 2.58 W to 2.38 W with increasing temperature from 10 °C to 50 °C.

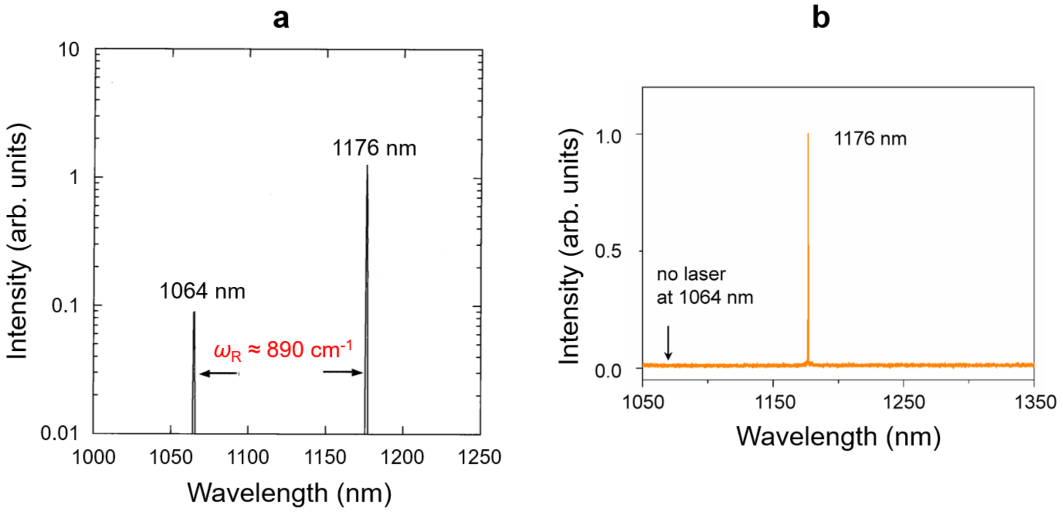

Figure S32. **a**, Laser spectrum of the actively Q-switched self-Raman laser of Nd:YVO_4 crystal[6]. **b**, Laser spectrum of our photon-phonon collaboratively pumped laser.

We compared our photon-phonon collaboratively pumped (PPCP) laser with self-Raman laser, see Fig. S33. It is observed that the slope efficiency of PPCP laser is significantly higher than that of traditional self-Raman lasing, owing to our PPCP laser is a direct phonon-triggering lasing, rather than nonlinear stimulated Raman scattering.

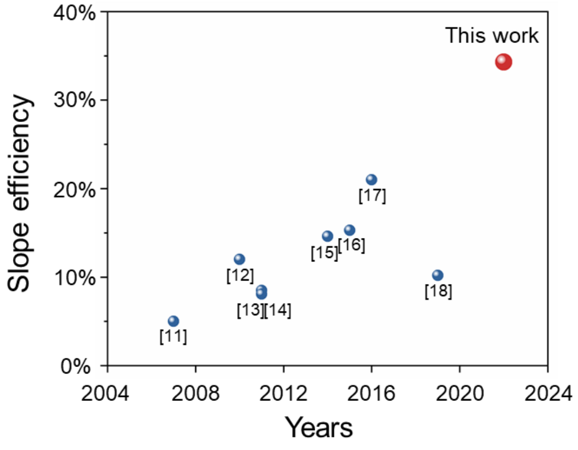

Figure S33. Comparison between Raman lasers of Nd:YVO_4 and our work[11-18].

In addition, modification of fluorescence spectrum by the cavity is also precluded. As shown in Fig. S34, there is no additional fluorescence peaks with the cavity coated on the Nd:YVO_4 crystal surfaces. Therefore, the lasing is not from the modification of fluorescence by cavity mirrors.

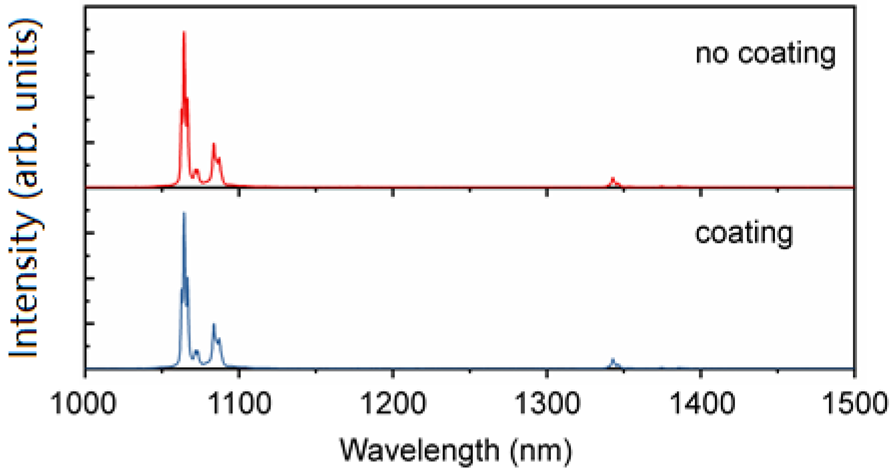

Figure S34. Fluorescence spectrum of uncoated and coated Nd:YVO_4 crystal.

Finally, we measured the temperature-dependent pump absorption in our lab. As shown in Fig. S35, the absorbed pump power gradually decreased with the increasing temperatures. The absorption efficiency reduced from 66.9% at 283 K to 61.3% at 353 K. This case is well consistent with previous report[19], where the absorption cross section of Nd:YVO_4 decreased from (58.6 ± 0.2) pm^2 at 291 K to (30.9 ± 0.6) pm^2 at 430 K. Therefore, the low-threshold of Nd:YVO_4 1176 nm laser at high temperature cannot be attributed to the increased pump absorption cross-section.

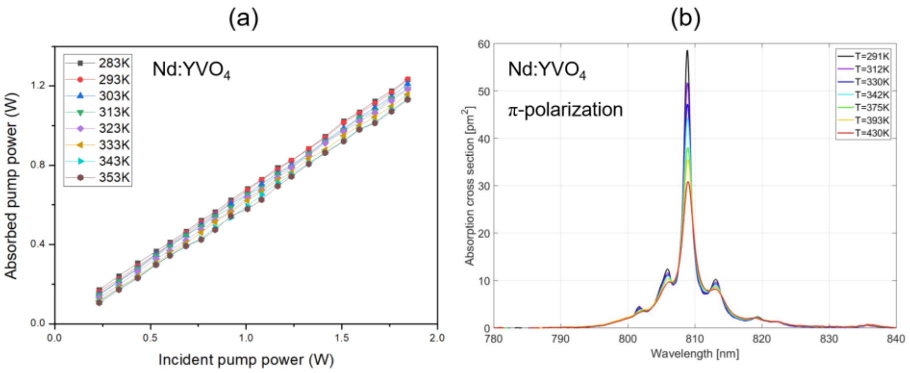

Figure S35. Temperature-dependent pump absorption of Nd:YVO_4 crystal. **a**, Absorbed pump power versus incident pump power under 283-353 K. **b**, Temperature-dependent absorption cross-section of Nd:YVO_4 crystal[19].

Section-III: Thermal effect in a four-energy level laser system

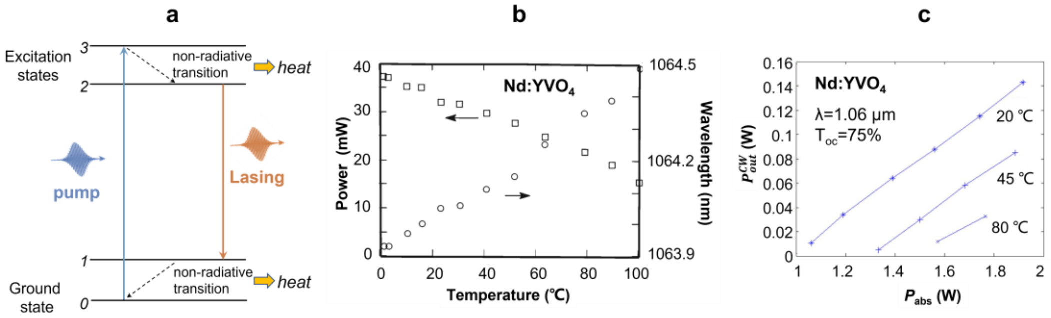

Figure S36. a, Schematic of a four-level Nd^{3+} laser system. **b**, Temperature-dependent laser performances of $Nd:YVO_4$ at 1064 nm[5,20]. **c**, Temperature-dependent laser performances of $Nd:YVO_4$ at 1064 nm[21].

Thermal effect is crucial in three-level lasers or quasi-three-level laser systems, *e.g.*, ruby laser and Yb:YAG laser, due to the temperature-dependent populations of electrons at lower levels. However, the thermal effect is also a crucial problem for four-energy levels (*e.g.*, Nd^{3+} ion) because their laser performances also deteriorated with increasing temperature (see Fig. S1 and Table S1).

A typical four-level Nd^{3+}-laser system is depicted in Fig. S36(a). In the presence of pumping light, the electrons on the ground state will be excited to the top one, then rapidly relaxed to the lower level 2 with non-radiative transitions. After the laser transition to terminal level 1, there exists another non-radiative transition between level 1 and ground level 0. Thus, two non-radiative transition processes brought serious thermal problems in traditional four-level laser systems. In laser theory, the population of level 1 can be considered as zero (large ΔE between 0 and 1). Therefore, if we don't consider electron-phonon coupling effect and thermally induced cross-relaxation, heat is not a big issue in Nd^{3+}-lasers because the population of level 1 was nearly independent on crystal temperature. As a result, the fluorescence lifetime and hence the power performance are quite insensitive to variations in temperature[22].

However, if the electron-phonon coupling effect was involved in the lasing process, the thermal effect would also be a great issue for four-level systems. With the increasing temperature, the electron-phonon coupling effect can modulate the energy transfer between electronic transition and lattice vibrations, thus leading to an inevitable influence on the population of phonon-involved levels. Therefore, thermal effect is an important topic in both three-level and four-level laser systems. In previous reports, the output power of $Nd:YVO_4$ microchip lasers at 1064 nm decreased with increasing temperature from 0 to 100 °C,

which is common for solid-state lasers. In addition, the lasing thresholds also increased at high temperatures.

In our work, we realized a thermally-enhanced 1176 nm laser with the assistance of hot phonons, where the temperature does change not only the stimulated emission cross-section but also the population inversion. This is a great forward step for laser physics.

Section-IV: The difference between alexandrite laser and Nd:YVO_4 laser

In previous reports, the output power increases while lasing threshold decreases at high temperatures. However, we must emphasize that our "phonon-pumped" Nd:YVO_4 laser is fundamentally different from those alexandrite lasers.

Indeed, some alexandrite lasers have an anomalous temperature dependence on laser threshold. we summarized the temperature-dependent alexandrite laser performances in Table S5. We also performed a similar alexandrite laser experiment in our lab. As shown in Fig. S37, the lasing threshold does indeed decrease with the increasing water-cooling temperatures, as well as higher output power. However, the laser wavelength inevitably has a significant red shift from 761 to 772 nm, which is vital evidence to show the difference.

Table S5. A summary of temperature-dependent alexandrite laser performances

Crystal	Pump source	Temperature range	Laser wavelength	Lasing threshold	Output power	Reference
0.4at.% Cr^{3+}:$BeAl_2O_4$ diameter:2.3mm length: 40mm	flash-lamp pulse: 104 μs	34-310 ℃	752-790 nm λ red shift at high temperature	first decrease, then increase; P_{th} is minimum at 225 ℃	P_{out} first increase, then decrease, P_{out} is maximum at 225℃	Opt. Lett. 7, 608 (1982)
c-cut 0.12at.% Cr^{3+}:$BeAl_2O_4$ diameter:6.35mm length: 96mm	flash-lamp pulse: 200 μs	15-64 ℃	λ ~ 750 nm	first decrease, then increase; P_{th} is minimum at 42 ℃	P_{out} increase at high temperature	IEEE J. Quantum Electron. 16, 1302 (1980)
c-cut 0.13at.% Cr^{3+}:$BeAl_2O_4$ diameter: 5mm length: 7mm	blue laser diode (λ=444 nm)	78-400 K	T<220K, λ=680 nm; T=260-400K, λ~745-751 nm λ red shift at high temperature	---	T<220K, P_{out} decrease at high temperature. T=260-400K, P_{out} first increase, then decrease. P_{out} is maximum at 350 K	Laser Phys. 27, 115801 (2017)
Brewster-cut 0.24at.% Cr^{3+}:$BeAl_2O_4$ diameter:4×4mm^2 length: 8mm	bule laser diode (λ=444 nm)	10-60 ℃	T=10-30 ℃ λ=762-760 nm; T=25-60 ℃ λ=774-772 nm. λ blue shift at high temperature	---	P_{out} decrease at high temperature	Opt. Express 31, 5832 (2023)
c-cut 0.2at.% Cr^{3+}:$BeAl_2O_4$ diameter: 4×4mm^2 length: 10mm	red laser diode (λ=638 nm)	10-50 ℃	λ=761-772 nm λ red shift at high temperature	P_{th} decrease at high temperature	P_{out} increase at high temperature	Our experimental results

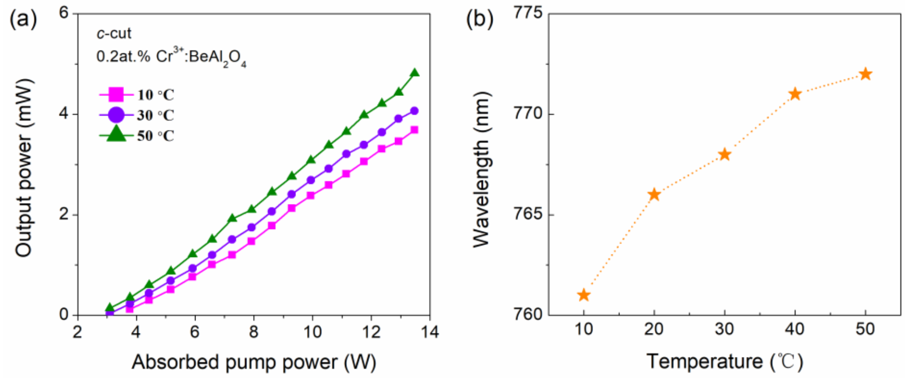

Figure S37. Temperature-dependent alexandrite lasers. **a**, output power, **b**, laser wavelength.

This anomalous temperature-dependent threshold of the alexandrite laser can be attributed to the Boltzmann distribution of ^2E and $^4T_2$ levels generated by the Stark splitting. Due to the long lifetime of 1.54 ms, a metastable electronic ^2E level provide the capacity for significant energy storage. The energy difference between ^2E and $^4T_2$ is 800 cm^{-1}. The resulting $^4T_2 \rightarrow ^4A_2$ transitions are natural at a finite temperature. At high temperatures, a successive thermal population of the $^4T_2$ level occurs in accordance with the Boltzmann distribution, where the thermal excitation rates for the $^2E \rightarrow ^4T_2$ transition increase, thus providing enhanced gain on the $^4T_2 \rightarrow ^4A_2$ vibronic laser transitions. This laser physical mechanism is plotted in Fig. S38.

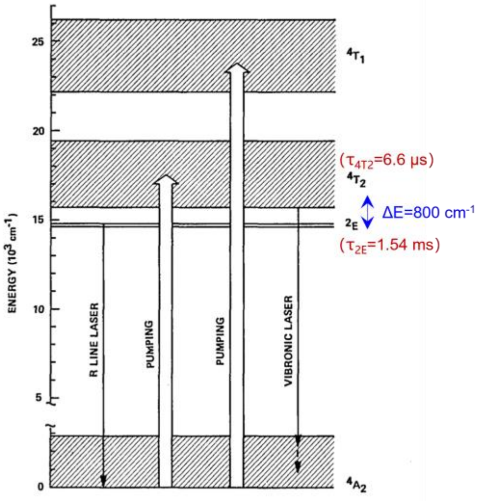

Figure S38. Energy-level diagram for alexandrite lasers. Thermal excitation from the ^2E storage level populates the $^4T_2$ state, which serves as the upper level for vibronic-laser operation.

Here, we must emphasize that our "phonon-pumped" $Nd:YVO_4$ laser is different from those alexandrite lasers. The alexandrite laser is *a traditional vibronic laser*. Its laser performance is

determined by the electron-phonon coupling induced spectral broadening. In comparison, our Nd:YVO_4 laser is *a phonon-pumped lasing* process accompanied by lattice phonon annihilation at high temperature. This is a prerequisite for lasing at 1176 nm and 1168 nm in Nd:YVO_4 crystal. This would be a new and universal physical mechanism for many solid-state lasers.

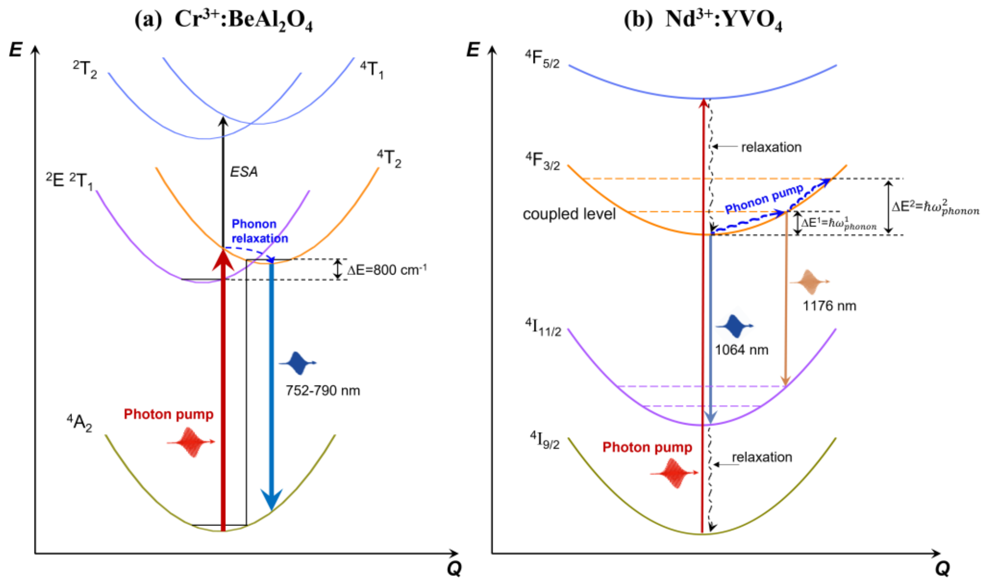

Figure S39. The configurational coordinate diagram of energy levels of Cr^{3+} ion and Nd^{3+} ion. **a**, alexandrite laser, **b**, Nd:YVO_4.

We used the configurational coordination model to show the difference. As shown in Fig. S39, the main differences include two aspects: (1) energy level difference ΔE, (2) laser low-level.

1. Energy level difference ΔE

Alexandrite is an intermediate crystal field material, in which ^2E and $^4T_2$ states are coupled. Their energy difference is 800 cm^{-1}, determined by the crystal field effect and independent of phonon energy. The lifetimes are 1.54 ms and 6.6 μs, respectively. The two *R* lines emitted from 2*E* occur in the vicinity of 680 nm, as for ruby. Because of the vibronic nature of the alexandrite laser, the fluorescence spectrum and corresponding gain cross-section become homogeneously broadening at high temperatures. Therefore, as the temperature increases, the gain of alexandrite increases, the gain peak shifts to a longer wavelength, and the fluorescence lifetime decreases. This temperature-dependent wavelength shift is a typical behavior of vibronic lasers, indicating the spectral broadening is a classical electron-phonon coupling effect with average participated phonon energy [$h\omega_{ph}$ ~524 cm^{-1}]. The laser wavelength is unable to control owing to the strong electron-phonon coupling effect and laser mode competition.

In Nd:YVO_4, the "phonon pumping" effect creates new vibronic levels in laser transitions. The quantized phonon energy determines the position of both initial and terminal levels for the laser emission. The population of phonon states follows the Bose-Einstein distribution. Therefore, its laser wavelength is determined by the phonon energy. Under A_{1g} mode phonon pumping ($h\omega_{ph}$ =891 cm^{-1}), its lasing wavelength maintain at 1176 nm without a large wavelength shift ($\Delta\lambda < 0.5$ nm at 296-338 K). Moreover, we can move it to 1168 nm by choosing E_g mode phonon-pumping ($h\omega_{ph}$ =839 cm^{-1}) via a birefringent filter. That is exactly why we call it a phonon-pumped laser, not a traditional vibronic laser.

2. Laser terminal level

In the alexandrite laser, the terminal laser level is a set of vibrational states well above the ground state. The laser initial level is a level 800 cm^{-1} above a long-lived storage level and in thermal equilibrium with it. The laser terminal level is very close to the ground-state level. Therefore, raising the temperature also tends to populate the terminal levels, especially those which lie closest to the ground level and which therefore correspond to the highest-energy (shortest-wavelength) photons. Since laser performance is highest with a maximally populated initial level and a minimally populated terminal level, it can be seen that increasing the temperature has two conflicting effects. As a result, there would be an optimal temperature T_m for alexandrite lasers, showing a typical V-shape temperature-dependent performance as demonstrated by Walling and Guch (**Supplementary Table S1**).

In comparison, the laser low-level of Nd:YVO_4 is $^4I_{11/2}$ level, not ground-state level $^4I_{9/2}$. There is a large energy difference of 1966 cm^{-1}. In general, the population of $^4I_{11/2}$ level can be neglected in the calculation of steady-state population inversion. As a result, our lasing threshold would continuously decrease with increasing temperatures in principle ($P_{th} = C/T_{th}$), until the intrinsic damage at high temperature. The minimum lasing threshold is determined by the thermal load capacity of the laser crystal and electron-phonon coupling intensity.

Section-V: Selection rules of phonons in electron-phonon coupling process

In this section, we analyzed the selection rules of lattice vibration modes involved in the phonon-triggered process. Symmetric consideration allows the determination of the vibronic activity with a specific mode. First, the vibronic-active phonon mode with different symmetry requires to be associated with a change of transition polarizability. Second, the phonon mode must be contained in the combination of the symmetries of electronic wave function and operator.

The significant difference in the rapidity of motion between electrons and nuclei allows us to apply the Born-Oppenheimer approximation to treat their correlated motions separately. Denoting the electron and lattice wave function of the system as $\varphi_e(R, r)$ and $\psi_v(R)$, respectively. The transition probability for the absorption or emission is given by the perturbation theory[23], see Equation (S1)

$$W_{i\to f} = Av \sum_{vf} \left| \langle \psi_{vf}\varphi_{ef} \middle| \widehat{M} \middle| \psi_{vi}\varphi_{ei} \rangle \right|^2 \delta\left(E - \left(E_f - E_i - n\hbar\omega_{\text{phonon}}\right)\right) \tag{S1}$$

where Av means the statistical average on initial phonon states ψ_{vi} with thermal distribution. $\langle \psi_{vf}\varphi_{ef} | \widehat{M} | \psi_{vi}\varphi_{ei} \rangle$ is the transition matrix element of the electric dipole moment from the initial state i to the final state f. The δ-function is introduced to maintain energy conservation. For simplicity and without loss of generality, we confine the vibronic coupling in the downward transitions process accompanied by phonon creation ($E_f < E_i$). It can be observed that the emitted photon energy is changed with the participation of phonons. Noting the incident radiation and lattice vibration modulate the electronic distribution in crystals together, and the distortion of electron clouds is related to the polarizability, the transition dipole moment in transition probability can be rewritten in the form of transition polarizability, see Equation (S2)

$$\langle \psi_{vf}\varphi_{ef} | \widehat{M} | \psi_{vi}\varphi_{ei} \rangle = \langle \psi_{vf}\varphi_{ef} | \widehat{P} | \psi_{vi}\varphi_{ei} \rangle \cdot \boldsymbol{E} \tag{S2}$$

where $\boldsymbol{E}$ represents the electric field of incident radiation field, which could induce the electronic redistribution. The transition polarizability $P_{if} = \langle \psi_{vf}\varphi_{ef} | \widehat{P} | \psi_{vi}\varphi_{ei} \rangle$ is a function of atomic displacement $\Delta R_{l,k}$, reflecting the electron cloud modulation induced by lattice vibration. Thus, we can expand transition polarizability in power a series of $\Delta R_{l,k}$ at the host lattice equilibrium configuration, see Equation (S3)

$$\begin{aligned} P_{if} &= P_{if}^{(0)} + P_{if}^{(1)} + \cdots \\ &= P_{if}^{(0)} + \sum_{l,k,\gamma} \left(\frac{\partial P_{if}}{\partial R_{l,k}^{\gamma}}\right)_0 \Delta R_{l,k}^{\gamma} + \frac{1}{2} \sum_{l,k,\gamma} \sum_{l',k',\delta} \left(\frac{\partial^2 P_{if}}{\partial R_{l,k}^{\gamma} \partial R_{l',k'}^{\delta}}\right)_0 \Delta R_{l,k}^{\gamma} \Delta R_{l',k'}^{\delta} + \cdots \end{aligned} \tag{S3}$$

We could replace lattice displacement $\Delta R_{l,k}$ in Eq. (S3) by complex normal coordinate $Q_r(\boldsymbol{q})$ (wave vector is $\boldsymbol{q}$), see Equation (S4)

$$P^{(1)}_{if,\alpha\beta} = N^{-1/2} \sum_{qr} P^{(1)}_{\alpha\beta}(\boldsymbol{q}, r) Q_r(\boldsymbol{q}) \tag{S4}$$

where N is the number of unit cells contained in the macroscopic crystal. The vibronic transition takes place only when the expansion coefficient $P^{(1)}_{\alpha\beta}(\boldsymbol{q}, r)$ is nonzero. According to Neumann's principle[24], the symmetry operations of any physical property of a crystal must include the symmetry operations of the crystal point group, hence indicate that transition polarizability can be transformed as the symmetry operations from crystal point group in two equivalent ways: by transforming the complex normal coordinates $Q_r(\boldsymbol{q})$ and by transforming the transition polarizability tensor $P^{(1)}_{if,\alpha\beta}$. Confining ourselves to the long-wavelength limit ($\boldsymbol{q}\rightarrow 0$) since vibronic laser wavelength is much larger than the lattice constant. One obtains the equation for determining the expansion coefficients, see Equation (S5)

$$P^{(1)}_{\varepsilon\eta}(0, r) = \sum_r \sum_{\alpha\beta} P^{(1)}_{\alpha\beta}(0, r) \frac{1}{g} \sum_R \left(R_{\alpha\varepsilon} R_{\beta\eta} D_r(R)\right) \tag{S5}$$

where $R_{\alpha\varepsilon,\beta\eta}$ are the components of rotation operation operating on tensor $P^{(1)}_{if,\alpha\beta}$, and $D_r(R)$ is the operation matrix operating on $Q_r(0)$, g is the number of rotation operations R. The expansion coefficient $P^{(1)}_{\varepsilon\eta}(0, r)$ is nonzero when $\frac{1}{g} \sum_R \left(R_{\alpha\varepsilon} R_{\beta\eta} D_r(R)\right) \neq 0$. It is worth noting that orthogonality theorem for irreducible representations[25], the symmetry of vibronic-activity phonon mode must be contained in the symmetry of transition polarizability tensors.

Besides, based on group theory, the matrix element is zero if the combination of the symmetry of initial wave functions and the operator does not contain the symmetry of final states wave functions[26]. As the symmetry of active ions doped in crystals is lower than that of the free ions, the degeneracy of electronic energy levels will be removed or partially removed, namely stark splitting effect. The splitting mainly relies on the symmetry of the coordination ligand containing active ions. It is a result of group theory that the representations of the rotation group are reduced into the irreducible representations of the point group, and this process is completely consistent with the splitting of the electronic energy levels. Thus, we can infer the irreducible representations of φ_{ei} and φ_{ef}. As for ψ_{vi} and ψ_{vf}, further classified according to their symmetry represented by irreducible representations of the crystal point group. The electric dipole operator is vector-like and transforms as the coordinates x, y, z, thus the

irreducible representation of ***M*** can be obtained. Finally, the selection rule for vibronic transition can be expressed as Equation (S6)

$$D(\varphi_{ef}) \otimes D(\hat{M}) \otimes D(\psi_{vi}) \otimes D(\varphi_{ei}) = D(\psi_{v_1 f}) \oplus D\left(\psi_{v_2 f}\right) \oplus \dots \quad \text{(S6)}$$

For example, the irreducible representations of the *n*th phonon mode, that is qualified, should be contained in the direct product of the irreducible representations φ_{ei}, φ_{ef}, *M*, and ψ_{vi}. This selection rule can be applied to determine which phonon modes are allowed by symmetry and are vibronic active.

YVO_4 crystal belongs to tetragonal $I4_1$/amd space group, where the Y^{3+} (doped Nd^{3+}) ions in the D_{2d} site reside between (VO_4) tetrahedral units. There are 24 atoms in a YVO_4 unit cell. Therefore, the irreducible representation of phonons in YVO_4 crystal is written as:

$$\Gamma = 10E_u+4A_{2u}+36E+B_{2u}+10E_g+A_{2g}+4B_{2g}+B_{1g}+A_{1u}+2A_{1g}+2B_{1u}$$

According to our selection rules, only A_{1g}, B_{1g}, B_{2g} and E_g modes can contribute to the phonon-pumping process. So, the percentage of special phonons that satisfy the symmetric condition for enhanced cross section is $\frac{17}{72}$.

Among them, two phonons, A_{1g} mode at 891 cm^{-1} and E_g mode at 839 cm^{-1}, represent the strongest coupling modes in phonon-pumping process, corresponding to laser wavelengths at 1176 nm and 1168 nm, respectively. We have demonstrated the laser oscillation at these two wavelengths in experiments. In addition, the coating parameters can also modulate the laser wavelengths by amplifying the specific phonon modes.

Section-VI: Theory for general threshold equation P_{th} = C/T_{th}

For a laser gain medium, the optical amplification by stimulated emission can be obtained when the laser gain (*G*) balances the cavity loss (*L*). Under small signal approximation[26], the gain is written as Equation (S7),

$$G = n_{21}\sigma \tag{S7}$$

where n_{21} is the inversion population and σ denotes the emission cross section.

On the one hand, the thermal equilibrium condition requires the population of electrons at the lower energy level must be larger than that at the upper energy level. Therefore, pump light energy is needed to produce population inversion of specific energy level, exciting electrons from the ground state to excitation state, given Equation (S8)

$$P_{pump} = n_{21}\eta\hbar\omega_{\mathrm{photon}}/t, \tag{S8}$$

where $\hbar\omega_{\mathrm{photon}}$ is the photon energy of pump light and η is the efficiency of non-radiation transition between two excited-levels. P_{pump} represents the pump power and *t* is time.

On the other hand, the emission cross section σ at wavelength λ_0 can be obtained from the fluorescence spectra, given Equation (S9)

$$\sigma = \frac{\lambda_0^2}{8\pi\tau n^2}F, \tag{S9}$$

where τ is fluorescence lifetime, *n* is the refractive index of the gain medium, and *F* is the lineshape function of fluorescence spectra. However, for our electron-phonon coupling system, the contribution to lineshape function does not only originate from pure electronic transition but also phonon-triggered transition. Considering the monochromatic of laser, we used the lineshape function obtained by Huang-Rhys theory under the single-frequency approximation[22], see Equation (S10)

$$F = |M_{ij}|e^{-S}\frac{S^p}{p!}, \tag{S10}$$

where $|M_{ij}|$ is the matrix element of the electric moment, *S* is Huang-Rhys factor, and *p* is the number of involved phonons. In Eq. (S10), under the weak coupling $S\rightarrow 0$ condition, the emission cross section can be written as

$$\sigma = \frac{\lambda_0^2}{8\pi\tau n^2}|M_{ij}|S, \tag{S11}$$

$$S = \frac{\omega_0\Delta_{ij}^2}{2\hbar}. \tag{S12}$$

We consider a single-phonon-involved transition (*p*=1). In the harmonic approximation, phonons act as

a one-dimensional harmonic oscillator. Here, Δ_{ij} represents the lattice shift of equilibrium positions associated with an electronic transition from state i to j, the relaxation energy can be expressed as $\frac{1}{2}m\omega_0^2\Delta_{ij}^2$. The lattice relaxation energy can be evaluated by phonon energy $\hbar\omega_0$. Thus, for a one-dimensional harmonic oscillator, the total phonon energy is proportional to k_BT, which makes the emission cross section σ is proportional to T/τ.

Combining the relations of $n_{21} \propto P_{pump}t$ and $\sigma \propto T/\tau$, one finds that gain can be expressed as

$$G = n_{21} \times \sigma \propto P_{pump}T \times t/\tau. \tag{S13}$$

As the laser gain G balances the loss C, the fluorescence lifetime τ becomes ineffective due to the stimulated radiation (lasing) process operating, it provides only a time unit to normalize that in the power unit. Substituting P_{pump} by P_{th} and T by T_{th}, we can get the relation for general threshold

$$P_{th} \times T_{th} \propto C. \tag{S14}$$

Meanwhile, as the gain G gradually approaches saturation with increasing pump power, the condition of steady state is reached, the output power P_{out} can be expressed as

$$P_{out} \propto \frac{1}{\sigma\tau}(\alpha\sigma\tau P_{pump} - \beta), \tag{S15}$$

where α and β represent the intrinsic parameters of lasing system, considering $\sigma \propto T/\tau$, we can obtain the relation between output power P_{out} and temperature T

$$P_{out} \propto \alpha P_{pump} - \frac{\beta}{T}. \tag{S16}$$

Section-VII: Steady-state rate equation for PPCP laser

We give a steady-state rate equation for our laser. The temperature-dependent behavior of PPCP laser can be described by the coupled rate equations. For our phonon-involved four-level system[27], the rate equations can be written as,

$$\frac{dn_{0'''}}{dt} = n_0 W_{00'''} - n_{0'''}(S_{0'''0''} + A_{0'''0})$$

$$\frac{dn_{0''}}{dt} = -n_{0''}W_{0''2'} - n_{0''}A_{0''2'} + n_{0'''}S_{0'''0''} - n_{0''}S_{0''1''}$$

$$\frac{dn_{1''}}{dt} = n_{0''}S_{0''1''} - n_{1''}W_{1''2'} - n_{1''}A_{1''2'}$$

$$\frac{dn_{2'}}{dt} = n_{0''}W_{0''2'} + n_{0''}A_{0''2'} - n_{2'}S_{2'0} + n_{1''}W_{1''2'} + n_{1''}A_{1''2'}$$

$$\frac{dn_0}{dt} = n_{2'}S_{2'0} - n_0 W_{00'''} + n_{0'''}A_{0'''0}$$

where n_i represents the electron population densities of level i, W_{ij} is the transition probability for stimulated radiation between level i and j, A_{ij} is the spontaneous transition probability, and S_{ij} is the nonradiative transition probability.

Considering the probability of nonradiative transition $S_{0'''0''}$ is much larger than spontaneous emission probability $A_{0'''0}$, and assuming a steady-state condition, $dn_{0'''}/dt$=0, we can obtain

$$n_0 W_{00'''} = n_{0'''}W_{0'''0''} = W_p$$

where W_p is pump rate.

For the case of thermal equilibrium, the relative populations of phonon-pumped level 1" and level 0" is determined by the Boltzmann distribution,

$$\frac{n_{1''}}{n_{0''}} = e^{-\frac{E_{1''}-E_{0''}}{k_B T}}$$

where $E_{1''}$-$E_{0''}$ is the energy separation between level 1" and 0", k_B is Boltzmann's constant, T is temperature. Considering $dn_{1''}/dt$= $dn_{0''}/dt$ =0, we can obtain the population inversion Δn as follows

$$\Delta n = n_{1''} - n_{2'} = \frac{W_p\left[S_{2'0} - e^{\frac{E_{1''}-E_{0''}}{k_B T}}\left(W_{0''2'} + A_{0''2'}\right) - \left(W_{1''2'} + A_{1''2'}\right)\right]}{\left[e^{\frac{E_{1''}-E_{0''}}{k_B T}}\left(2S_{0''1''} + W_{0''2'} + A_{0''2'}\right) - \left(W_{1''2'} + A_{1''2'}\right)\right]S_{2'0}}$$

Where the population of lasing up-level $n_{1''}$ is

$$n_{1''}=\frac{W_p}{e^{\frac{E_{1''}-E_{0''}}{k_BT}}\left(W_{0''2'}+A_{0''2'}+2S_{0''1''}\right)-\left(W_{1''2'}+A_{1''2'}\right)},$$

and the population of lasing low-level $n_{2'}$ is

$$n_{2'}=\frac{W_p\left[e^{\frac{E_{1''}-E_{0''}}{k_BT}}\left(W_{0''2'}+A_{0''2'}\right)+\left(W_{1''2'}+A_{1''2'}\right)\right]}{S_{2'0}\left[e^{\frac{E_{1''}-E_{0''}}{k_BT}}\left(W_{0''2'}+A_{0''2'}+2S_{0''1''}\right)-\left(W_{1''2'}+A_{1''2'}\right)\right]}.$$

It clearly shows the relation between population inversion with the temperature, higher temperature *T* is helpful for the formation of population inversion. At the condition of steady state, the population inversion stabilized and the electron population densities at each level does not change with time. However, before the creation of population inversion, population inversion would change with time and our model is suitable for this condition.

Section-VIII: A comparison between solid-state lasers, semiconductor lasers and PPCP laser.

Table S6. A comparison for solid-state lasers and semiconductor lasers.

		pump wavelength λ_p (nm)	temporal characteristics	slope efficiency	optical-to-optical efficiency	reference
PPCP laser	$Nd:YVO_4$ PPCP laser at 1176 nm	808	cw	34.3%	17.7%	this work
LD-pumped self-Raman laser	$Nd:YVO_4$ self-Raman laser at 1176 nm	808	cw	8.1%	7.7%	Chin. Phys. Lett. 28, 054202 (2011)
		808	pulse (ns)	19.2%	13.9%	Opt. Lett. 29, 1915-1917 (2004)
		808	pulse (ns)	--	18.2%	Laser Phys. Lett. 6, 26-29 (2009)
		808	pulse (ps)	8.2%	5.3%	Opt. Laser Technol. 89, 1-5 (2017)
		878.9	cw	21%	20%	Chin. Phys. B. 25, 114207 (2016)
		880	cw	8.98%	7.3%	Opt. Lett. 40, 3524-3527 (2015)
		878.6	quasi-cw	--	28.6%	Infrared and Laser Engineering 50, 20200227 (2021)
		880	quasi-cw	15.3%	11.7%	Proc. SPIE 9347, 934718 (2015)
LD-pumped self-Raman laser	$Nd:YVO_4+YVO_4$ laser at 1176 nm	808	cw	8.5%	7.8%	Appl. Phys. B 103, 559-562 (2010)
	$YVO_4+Nd:YVO_4+YVO_4$ laser at 1176 nm	808	cw	14.6%	13.3%	Acta. Phys. Sin. 63, 154208 (2014)
	$YVO_4+Nd:YVO_4+YVO_4$ laser at 1176 nm	808	ns	16.3%	12.4%	Appl. Phys. B 101, 743-746 (2010)
Optically-pumped Raman laser	$Nd:YAG+YVO_4$ Raman laser at 1176 nm	808	pulse (ns)	--	8.8%	J. Phys. D: Appl. Phys. 50, 465303 (2017)
	diamond Raman laser at 1178 nm	1018	cw	38%	24.4%	Appl. Phys. Lett. 121, 141104 (2022)
Optically-pumped solid-state laser	Nd:YAG-SHG pumped Ti:sapphire laser at 1178 nm	532	pulse (ns)	--	0.48%	Conference on Lasers and Electro-Optics. 14, 296-298 (1987)
	LD-pumped Yb:LCB laser at 1162 nm	976	cw	8.4%	7%	Light Sci. & Appl. 12, 203 (2023)
	LD-pumped Yb:YCOB laser at 1200 nm	976	cw	16.4%	15%	Opt. Lett. 48, 4913-4916 (2023)
Optically-pumped semiconductor laser	VECSEL laser at 1180 nm	808	cw	33%	27.3%	Electronics Letters 49, 59-60 (2013)
	VECSEL laser at 1178 nm	808	cw	29.4%	27%	Proc. SPIE 9349, 93490U (2015)
	VECSEL laser at 1185.5 nm	808	cw	38%	29%	Electronics Letters 54, 1135-1137 (2018)
Electrically - pumped semiconductor laser	semiconductor DBR laser at 1180 nm	--	cw	--	electrical-to-optical efficiency = 31%	Opt. Lett. 41, 657-660 (2016)

Here, we give a comprehensive comparison for conversion efficiency of many solid-state lasers. As listed in **Table S6**, among LD-pumped solid-state lasers at 1176 nm, our PPCP laser exhibits the highest slope efficiency at 1176 nm. In addition, compared to the state-of-art diamond Raman laser at 1178 nm, our PPCP laser also has a comparable slope efficiency. Moreover, our laser efficiency is also comparable to optically-pumped (or electrically-pumped) semiconductor laser at 1180 nm. Therefore, our PPCP laser doesn't exist a penalty in efficiency, but a comparable (even better) efficiency to other solid-state lasers.

Supplementary References

1. Miller, S. A., Caspers, H. H. and Rast, H. E. Lattice vibrations of yttrium vanadate. *Phys. Rev.* **168**, 964-969 (1968).

2. Watanabe, K., Taniguchi, T. and Kanda, H. Direct-bandgap properties and evidence for ultraviolet lasing of hexagonal boron nitride single crystal. *Nat. Mater.* **3**, 404-409 (2004).

3. Holder, C. O. et al. Nonpolar III-nitride vertical-cavity surface emitting lasers with a polarization ratio of 100% fabricated using photoelectrochemical etching. *Appl. Phys. Lett.* **105**, 031111 (2021).

4. Loiko, P. et al. Watt-level europium laser at 703 nm. *Opt. Lett.* **46**, 2702-2705 (2021).

5. Bowkett, G. C. Nd:YVO_4 microchip lasers and amplifiers, Victoria University of Technology, PhD thesis, Page 118 (1999).

6. Chen, Y. F. High-power diode-pumped actively Q-switched Nd:YVO_4 self-Raman laser: influence of dopant concentration. *Opt. Lett.* **29**, 1915-1917 (2004).

7. Fan, L. et al. Efficient continuous-wave eye-safe Nd:YVO_4 self-Raman laser at 1.5 μm. *Opt. Lett.* **46**, 3183-3186 (2021).

8. Wan, X. B. et al. Comparison of c-Nd:YVO_4/YVO_4 Raman Lasers and c-Nd:YVO_4 Self-Raman Lasers. *Laser Phys.* **22**, 106-110 (2012).

9. Zverev, P. G. The influence of temperature on Raman modes in YVO_4 and $GdVO_4$ crystal. *J. Phys.: Conf. Ser.* **92**, 012073 (2007).

10. Ding, X. et al. Efficient eye-safe Nd:YVO_4 self-Raman laser in-band pumped at 914 nm. *IEEE Photonics J.* **7**, 1503807 (2016).

11. Burakevich, V. N. et al. Diode-pumped continuous-wave Nd:YVO_4 laser with self-frequency Raman conversion. *Appl. Phys. B.* **86**, 511-514 (2007).

12. Fan, L. et al. A compact efficient continuous-wave self-frequency Raman laser with a composite YVO_4/Nd:YVO_4/YVO_4 crystal. *Appl. Phys. B.* **101**, 493-496 (2010).

13. Zhu, H. Y. et al. Efficient continuous-wave YVO_4/Nd:YVO_4 Raman laser at 1176 nm. *Appl. Phys. B.* **103**, 559-562 (2011).

14. Zhu, H. Y. et al. Compact continuous-wave Nd:YVO_4 laser with self-Raman conversion and sum frequency generation. *Chin. Phys. Lett.* **28**, 054202 (2011).

15. Fan, L. et al. Laser diode end-pumped continuous-wave Nd:YVO_4 self-Raman laser at 1175 nm. *Acta.*

Phys. Sin. **63**, 154208 (2014).

16. Kores, C. C. et al. Diode-side-pumped continuous wave Nd^{3+} : YVO_4 self-Raman laser at 1176 nm. *Opt. Lett.* **40**, 3524-3527 (2015).

17. Fan, L. et al. An efficient continuous-wave YVO_4/Nd:YVO_4/YVO_4 self-Raman laser pumped by a wavelength-locked 878.9 nm laser diode. *Chin. Phys. B.* **25**, 114207 (2016).

18. Fan, L. et al. Multi-wavelength continuous-wave Nd:YVO_4 self-Raman laser under in-band pumping. *Chin. Phys. B.* **28**, 084210 (2019).

19. Silvia Cante, Towards the power-scaling of sub-1 μm cryogenically cooled lasers, University of Southampton, PhD thesis (2021).

20. Qiu, M. X. et al. Performance of a Nd:YVO_4 microchip laser with continuous-wave pumping at wavelengths between 741 and 825 nm. *Appl. Phys.* **32**, 2085-2086 (1993).

21. Delen, X. et al. Temperature dependence of the emission crosssection of Nd:YVO_4 around 1064 nm and consequences on laser operation. *J. Opt. Soc. Am. B* **28**, 972-976 (2011).

22. Sennaroglu, A. Efficient continuous-wave operation of a diode-pumped Nd:YVO_4 laser at 1342 nm. *Opt. Commun.* **164**, 191-197 (1999).

23. Huang, K. & Rhys, A. Theory of light absorption and non-radiative transitions in F-centers. *Proc. Roy. Soc. A: Math., Phys. Eng. Sci.* **204**, 406-423 (1950).

24. Shi, P. P., et al. Symmetry breaking in molecular ferroelectrics. *Chem. Soc. Rev.* **45**, 3811-3827 (2016).

25. Burns, G. *Introduction to Group Theory with Applications* (Academic Press, 1977).

26. Evans, B. D. & Kemp, J. C. Vibronic aspects of the CaO and MgO F bands. *Phys. Rev. B* **2**, 4179-4189 (1970).

27. Koechner, W. *Solid-State Laser Engineering* (Springer, 1976).
