# Supplementary material for: Photon-phonon collaboratively pumped laser
Source: Nat Commun. 2023 Dec 7;14:8110. doi: 10.1038/s41467-023-43959-9 (PMC10703827; doi:10.1038/s41467-023-43959-9)
Supplement: Supplementary file 3 — Reporting Summary [file 41467_2023_43959_MOESM3_ESM.pdf]

Corresponding author(s): Yan-Feng Chen Email: yfchen@nju.edu.cn

Lasing Reporting Summary

Nature Research wishes to improve the reproducibility of the work that we publish. This form is intended for publication with all accepted papers reporting claims of lasing and provides structure for consistency and transparency in reporting. Some list items might not apply to an individual manuscript, but all fields must be completed for clarity.

For further information on Nature Research policies, including our data availability policy, see Authors & Referees.

Experimental design

Please check: are the following details reported in the manuscript?

1. Threshold

Plots of device output power versus pump power over a wide range of values indicating a clear threshold

☒ Yes ☐ No

Main text Page 9, Figure 5c; Extended data Page 13-14, Table S4, Figure S29

2. Linewidth narrowing

Plots of spectral power density for the emission at pump powers below, around, and above the lasing threshold, indicating a clear linewidth narrowing at threshold

☒ Yes ☐ No

Main text Page 8; Extended data Page 8, Figure S13-S17

Resolution of the spectrometer used to make spectral measurements

☒ Yes ☐ No

The spectral resolution is 0.02 nm; see the Figure caption of Extended data Figure S11

3. Coherent emission

Measurements of the coherence and/or polarization of the emission

☒ Yes ☐ No

Extended data Page 9, Figure S18

4. Beam spatial profile

Image and/or measurement of the spatial shape and profile of the emission, showing a well-defined beam above threshold

☒ Yes ☐ No

Extended data Page 10, Figure S13-9-S26 Table S2

5. Operating conditions

Description of the laser and pumping conditions
Continuous-wave, pulsed, temperature of operation

☒ Yes ☐ No

Continuous-wave laser, see Extended data Page 11, Figure S27

Threshold values provided as density values (e.g. W cm^{-2} or J cm^{-2}) taking into account the area of the device

☒ Yes ☐ No

see Main text Page 9, Figure 5c; Table S4

6. Alternative explanations

Reasoning as to why alternative explanations have been ruled out as responsible for the emission characteristics
e.g. amplified spontaneous, directional scattering; modification of fluorescence spectrum by the cavity

☒ Yes ☐ No

We claim our photon-phonon collaboratively pumping mechanism, see Main text Page 3-4, Figure 1; Extended data Page 29-31, Figure S32

7. Theoretical analysis

Theoretical analysis that ensures that the experimental values measured are realistic and reasonable
e.g. laser threshold, linewidth, cavity gain-loss, efficiency

☒ Yes ☐ No

We confirmed our novel laser is reasonable, see Main text Page 10, Analytical theory; Extended data Page 41-42

8. Statistics

Number of devices fabricated and tested

☒ Yes ☐ No

We tested 5 crystals for photon-phonon collaboratively pumped laser, see Extended data Table S4 and Figure S29

Statistical analysis of the device performance and lifetime (time to failure)

☒ Yes ☐ No

Every laser device was tested for more than 5 times, see the error bars in Figure S29
